# Modeling for influenza vaccines and adjuvants profile for safety prediction system using gene expression profiling and statistical tools

**DOI:** 10.1371/journal.pone.0191896

**Published:** 2018-02-06

**Authors:** Eita Sasaki, Haruka Momose, Yuki Hiradate, Keiko Furuhata, Mamiko Takai, Hideki Asanuma, Ken J. Ishii, Takuo Mizukami, Isao Hamaguchi

**Affiliations:** 1 Department of Safety Research on Blood and Biological Products, National Institute of Infectious Diseases, Musashi-Murayama, Tokyo, Japan; 2 Influenza Virus Research Center, National Institute of Infectious Diseases, Musashi-Murayama, Tokyo, Japan; 3 Laboratory of Adjuvant Innovation, National Institutes of Biomedical Innovation, Health and Nutrition, Ibaraki, Osaka, Japan; 4 Laboratory of Vaccine Science, WPI Immunology Frontier Research Center, Osaka University, Suita, Osaka, Japan; Instituto Butantan, BRAZIL

## Abstract

Historically, vaccine safety assessments have been conducted by animal testing (*e*.*g*., quality control tests and adjuvant development). However, classical evaluation methods do not provide sufficient information to make treatment decisions. We previously identified biomarker genes as novel safety markers. Here, we developed a practical safety assessment system used to evaluate the intramuscular, intraperitoneal, and nasal inoculation routes to provide robust and comprehensive safety data. Influenza vaccines were used as model vaccines. A toxicity reference vaccine (RE) and poly I:C-adjuvanted hemagglutinin split vaccine were used as toxicity controls, while a non-adjuvanted hemagglutinin split vaccine and AddaVax (squalene-based oil-in-water nano-emulsion with a formulation similar to MF59)-adjuvanted hemagglutinin split vaccine were used as safety controls. Body weight changes, number of white blood cells, and lung biomarker gene expression profiles were determined in mice. In addition, vaccines were inoculated into mice by three different administration routes. Logistic regression analyses were carried out to determine the expression changes of each biomarker. The results showed that the regression equations clearly classified each vaccine according to its toxic potential and inoculation amount by biomarker expression levels. Interestingly, lung biomarker expression was nearly equivalent for the various inoculation routes. The results of the present safety evaluation were confirmed by the approximation rate for the toxicity control. This method may contribute to toxicity evaluation such as quality control tests and adjuvant development.

## Introduction

Vaccines are administered to a large population of healthy individuals including children and infants. Therefore, their safety and quality must be assured by national regulatory authorities and they require high levels of safety and lot-to-lot quality consistency to meet strict regulatory guidelines [1]. In addition to preclinical safety studies, the abnormal toxicity test (ATT) is conducted to evaluate general toxicity and the quality of lot-to-lot or batch-to-batch vaccines as part of post-licensing safety testing [2]. Some specific toxicity tests, such as the residual toxicity test for toxoids and neurovirulence test for live attenuated vaccines, are employed. Although these traditional toxicity tests are conducted *in vivo* using animals to ensure vaccine safety and, in part, provide a safety reference for reactions in humans, these methods have some limitations in that the results cannot be extrapolated for factors such as body weight loss and leukocyte reduction related to adverse events. Remarkably, these test methods have not changed for over 40 years. Thus, there is a need to update the current system of toxicological testing, and recent advances in omics technologies may be useful for achieving this goal. Advanced imaging and omics technologies (e.g., genomics, proteomics, and metabolomics) have been applied in toxicological analyses with robotized testing platforms that enable the toxicities of large numbers of substances to be tested in animal models and cell lines or with *in silico* computational methodologies using high-throughput screening. These techniques have shortened the testing process period and enabled determination of how chemicals interact with biological systems *in vivo*. However, it remains difficult to integrate advanced omics technologies into preclinical and post-licensure tests to evaluate vaccine safety.

To integrate the use of omics technologies with current testing methods, we performed comprehensive gene expression analysis in diphtheria tetanus and pertussis combined vaccine-treated rats [3] and successfully identified novel biomarkers for evaluating vaccine toxicity [4]. We next focused on the influenza vaccine because of its wider variation and cutting-edge vaccine development platform. The seasonal influenza vaccine was first developed as a formaldehyde-inactivated, whole virion inactivated vaccine (WPv) in hen’s eggs. Despite the high immunogenicity of WPv, adverse events including site reactions and fever were reported and, consequently, the split vaccine was introduced in the 1970s. Recently, many adjuvants have been developed to induce immunogenicity for pandemic influenza and several new administration routes, including intranasal and patched administration methods, have been assessed. Subcutaneous injection has been adopted for hepatitis B, rabies, and influenza vaccines [5–7]. Some vaccines have been injected via the intramuscular route, particularly aluminum hydroxide (alum) or oil-water emulsion-based adjuvanted vaccines [8,9]. Intranasal administration potentially induces mucosal immunity by dominantly inducing IgA production compared to subcutaneous vaccination, which shows high protective effects against the early mucosal infection stage [10,11]. Thus, choosing an appropriate vaccination route is important for maximizing the protective effects of vaccination [12]. It has been suggested that the vaccination administration route affects the potential toxicity of a vaccine formulation [13–15]. In preclinical tests, safety assessment of the dosing route used for human clinical use has been recommended [16]. Thus, a more robust safety assessment system capable of supporting several local vaccination routes (e.g., intranasal, intramuscular) is needed.

In Japan, the ATT was added to the guidelines for lot release testing of influenza vaccines. The leukocyte toxicity test (LTT) [2] is also used to assess the immunotoxicity of influenza vaccines. To integrate omics technology into the ATT for influenza vaccine safety, we comprehensively performed gene expression analysis in a subvirion influenza vaccine (HAv), WPv, and adjuvanted pandemic H5N1 influenza vaccine-treated rat model to identify novel biomarkers for evaluating vaccine toxicity [17]. The lung biomarker used in this study was identified in a study that collected all major organs such as the lung, liver, spleen, and blood of rats after inoculation with influenza vaccine followed by microarray analyses in each organ [17]. In the study, the lung showed the clearest clustering between the whole virion inactivated influenza vaccine (toxicity control) and split influenza vaccine (safety control) compared to the spleen, liver, and blood. Therefore, we carried out safety assessment of influenza vaccine by measuring gene expression changes in the lung. We also proposed a vaccine safety evaluation concept based on a systematic vaccinological approach and 18 lung-expressed biomarkers for evaluating the batch-to-batch consistency and safety of the seasonal influenza vaccine [17]. The expression levels of the 18 biomarkers were increased in the lung when WPv was injected intraperitoneally into rats, in contrast to HAv [17]. Therefore, the expression of these 18 biomarkers may in part reflect the biological effects of the vaccine, including adverse reactions to WPv. We also recently showed that the toxicity of adjuvanted influenza vaccines can be assessed even when administered by intranasal vaccination in mice using our lung expression-specific 18-biomarker gene set [18]. Therefore, this gene set can covers not only the intraperitoneal route, but also other routes.

The final aim of our long-term investigations is to provide a new and validated system for evaluating influenza vaccine and adjuvant safety using biomarkers considering the administration route. Few studies have examined how to derive conclusions by evaluating toxicity and safety information via changes in biomarkers such as biomarker gene expression levels. Therefore, we performed risk assessment on a formulation known to be toxic to humans (*i*.*e*., WPv and Poly I:C) as a vaccine-adjuvant toxicity control. To objectively and quantitatively evaluate the level of toxicity via biomarker changes, robust statistical methods must be developed. A new high-throughput evaluation method based on quantitative safety data should be developed to overcome these issues.

Here, we developed and validated a practical safety assessment system for vaccines and adjuvants using logistic regression analysis and biomarkers that can be used to evaluate various inoculation routes. AddaVax^™^ and poly I:C were used as model adjuvants and influenza vaccines were used as model vaccines, as the toxicity of the reference vaccine is available [2] and poly I:C is known to induce immunotoxicity in humans and animals [19–22]. First, we acquired data relating to body weight changes, white blood cell (WBC) counts, and biomarker gene expression levels in the lungs of vaccine-treated mice according to the ATT and LTT methods [2]. The vaccine formulations used in this study are listed in Table 1. Second, data acquired were used for logistic regression analysis to assess the predictability of the biomarkers and to develop a regression equation for safety classification. Finally, we carried out zone classification of the safety evaluation by analyzing gene expression levels.

**Table 1:** Influenza vaccine formulations used in study.

Abbreviations	Vaccine formulation	Antigen	Antigen contents (/mouse)	Adjuvant	Adjuvant content (/mouse)	Safety classification*
SA	-	-	-	-	-	safe
HA	Influenza split-product vaccine	A/New Caledonia/20/99; H1N1	10 μg HA strain/mouse	-	-	safe
HA + AddaVax 12.5%	Influenza split-product vaccine	A/New Caledonia/20/99; H1N1	10 μg HA strain/mouse	Addavax	12.5%	safe/low toxicity
HA + AddaVax 25%	Influenza split-product vaccine	A/New Caledonia/20/99; H1N1	10 μg HA strain/mouse	Addavax	25%	safe/low toxicity
HA + AddaVax 50%	Influenza split-product vaccine	A/New Caledonia/20/99; H1N1	10 μg HA strain/mouse	Addavax	50%	safe/low toxicity
HA + AddaVax 75%	Influenza split-product vaccine	A/New Caledonia/20/99; H1N1	10 μg HA strain/mouse	Addavax	75%	safe/low toxicity
HA + Poly I:C 1	Influenza split-product vaccine	A/New Caledonia/20/99; H1N1	10 μg HA strain/mouse	Poly I:C	1 μg	low toxicity/toxicity
HA + Poly I:C 5	Influenza split-product vaccine	A/New Caledonia/20/99; H1N1	10 μg HA strain/mouse	Poly I:C	5 μg	low toxicity/toxicity
HA + Poly I:C 10	Influenza split-product vaccine	A/New Caledonia/20/99; H1N1	10 μg HA strain/mouse	Poly I:C	10 μg	low toxicity/toxicity
HA + Poly I:C 20	Influenza split-product vaccine	A/New Caledonia/20/99; H1N1	10 μg HA strain/mouse	Poly I:C	20 μg	low toxicity/toxicity
RE	Whole-virion inactivated influenza vaccine	A/Newcaledonia/20/99 (H1N1), A/Hiroshima/52/2005 (H3N2), and B/Malaysia/2506/2004	10 μg HA strain/mouse	-	-	toxicity

Safety classifications were set based on the presence or absence of side reactions such as fever with reference to clinical report literature [19–22].

## Materials and methods

### Animals and ethics statement

Female 6–7-week-old BALB/c mice (16–20 g) were obtained from SLC (Tokyo, Japan). All mice were housed in rooms maintained at 23 ± 1°C and 50 ± 10% relative humidity, under a 12 h light/dark cycle, and provided with food and water *ad libitum*. The mice were acclimated for at least two days before commencing the experiments. All animal experiments were performed according to institutional guidelines and with the approval of the National Institute of Infectious Diseases Animal Care and Use Committee (Permit Number: 115130).

### Vaccines and adjuvants

All vaccine and adjuvant solutions were individual 0.5-mL doses for intraperitoneal injection, 0.1-mL doses for intramuscular injection, and 30-μL doses for intranasal injection. Therefore, the concentrations of the vaccine and adjuvant in the formulations were appropriate for their inoculation routes, as the inoculation volume per dose differs for each inoculation route. The vaccine formulations used in this study are listed in Table 1. The toxicity reference for the national RE vaccine was issued by NIID (Japan) [2]. RE is a lyophilized whole-virion preparation of an inactivated influenza virus comprising three different types of inactivated whole-virion components: A/Newcaledonia/20/99 (H1N1), A/Hiroshima/52/2005 (H3N2), and B/Malaysia/2506/2004. RE was used as the toxicity reference in the LTT in Japan [2]. This sample was reconstituted in 12 mL of saline (SA) for intraperitoneal injection, 2.4 mL for intramuscular injection, and 720 μL for intranasal administration, prepared at 1 U per mouse for each inoculation route. The influenza A virus (A/New Caledonia/20/99; H1N1) split-product vaccine (HAv) was kindly provided by the Kitasato Institute (Tokyo, Japan). HAv was reconstituted in an appropriate volume of SA and serially diluted with SA to prepare solutions of 10 μg HA/mouse for any inoculation route. Poly I:C, purchased from Sigma-Aldrich (St. Louis, MO, USA), was reconstituted in an appropriate volume of HAv solution to give concentrations of 5, 10, and 20 μg poly I:C per dose. These dosing amounts are known as sufficient volumes to be effective in mice and are equivalent to the human dose [19, 20]. AddaVax^™^ (InvivoGen, San Diego, CA, USA) is squalene-based oil-in-water emulsion adjuvant similar to MF59 [21]. MF59 is a safe and potent adjuvant for use with human vaccines, with more than 27 million doses already administered to humans [22]. Therefore, we used AddaVax^™^ as an adjuvant because of its safety and effectiveness. MF59 is inoculated with the vaccine in a volume of 50% v/v [21]; thus, we prepared 25, 50, and 75% v/v doses of the AddaVax^™^-containing vaccine. However, for nasal inoculations only, 12.5, 50, and 50% v/v samples of AddaVax^™^ were prepared. Nasal inoculation requires a high concentration of HAv because it uses small administration volumes (30 μL/mouse). Additionally, because AddaVax^™^ is a liquid formulation, the HAv concentration is diluted when combined with AddaVax^™^. Thus, the blending ratio of AddaVax^™^ was limited to 50% for nasal inoculation.

### ATT and LTT

ATT was performed according to the Minimum Requirements for Biological Products (MRBP) [2], with slight modifications for using mice as the test animals. After the bodyweights of the 7-week-old female BALB/c mice were measured, the mice were administered SA, HAv, PolyI:C-adjuvanted HAv, AddaVax^™^-adjuvanted HAv, or RE by various inoculation routes. Mouse bodyweights were measured again 16 h after administrating the compounds. LTT was performed according to the MRBP [2] with slight modifications. Briefly, at 16 h post-vaccination, the mice were anesthetized with pentobarbital. Their blood was collected via the inferior vena cava and immediately transferred into EDTA-coated tubes. WBC and platelet numbers were counted with a Celltac MEK-6450 automatic hemocytometer (Nihon Kohden, Tokyo, Japan).

### Lung lysate preparation and QuantiGene Plex (QGP) assays

Lung lysates were prepared and the QGP assay was performed as described in our previous studies [18,23,24]. Briefly, mouse lungs were collected 16 h after the vaccine and/or adjuvant was administered. The organs were immediately stored in RNAlater (Thermo Fisher Scientific, Waltham, MA, USA) and then homogenized before the QGP assay was performed according to the QuantiGene Plex Reagent System instructions (Panomics/Affymetrix, Santa Clara, CA, USA) as described previously [18,23,24]. Probes for 18 of the 20 biomarkers were designed (Table 2). Two genes, *beta-2 microglobulin* and *Npc1*, could not be designed for mice, as the probes are not optimized for sequence specificity and their absolute expression levels were low compared to those of the other biomarkers [18]. Because QGP detects biomarker gene expression simultaneously, its ability to measure gene expression levels is limited. Thus, genes expressed at extremely low levels are considered out of range in QGP analysis and are therefore not detectable. Consequently, these two genes were excluded from this study. Gene expression was assessed in the QGP assay by luminescence, which was measured with a Bio-Plex 200 System (Bio-Rad, Hercules, CA, USA). The expression levels of the biomarkers and β-actin were quantified simultaneously, and the expression ratio of each biomarker gene to that of β-actin was calculated. For logistic regression analyses, the expression levels of the 18 genes were converted into relative expression levels as compared with the SA-treated groups before the analyses. These conversions were performed to eliminate differences in the fluorescence intensity values for QGP analysis among the individual experiments.

**Table 2:** Biomarkers used for the safety evaluation of influenza vaccines.

Symbol	Official Full Name	Accession
***Cxcl11***	Chemokine (C-X-C motif) ligand 11	NM_019494
***Cxcl9***	**Chemokine (C-X-C motif) ligand 9**	**NM_008599**
***Zbp1***	**Z-DNA binding protein 1**	**NM_021394**
***Mx2***	**MX dynamin-like GTPase 2**	**NM_013606**
***Irf7***	**Interferon regulatory factor 7**	**NM_016850**
***Lgals9***	**Lectin, galactoside-binding, soluble, 9**	**NM_010708**
***Ifi47***	**Interferon gamma inducible protein 47**	**NM_008330**
***Tapbp***	**TAP binding protein (tapasin)**	**NM_001025313**
***Csf1***	**Colony stimulating factor (macrophage)**	**NM_007778**
***Timp1***	**Tissue inhibitor of metalloproteinase 1**	**NM_001044384**
***Trafd1***	**TRAF type zinc finger domain containing 1**	**NM_001163470**
***Lgals3bp***	**Lectin, galactoside-binding, soluble, 3 binding protein**	**NM_011150**
***Psmb9***	**Proteasome (prosome, macropain) subunit, beta type, 9**	**NM_013585**
***C2***	**Complement component 2**	**NM_013484**
***Tap2***	**Transporter 2, ATP-binding cassette, sub-family B (MDR/TAP)**	**XM_006525355**
***Ifrd1***	**Interferon-related developmental regulator 1**	**NM_013562**
***Psme1***	**Proteasome (prosome, macropain) activator subunit 1**	**NM_011189**
***Ngfr***	**Nerve growth factor receptor**	**NM_033217**

### Statistical analyses

For multiple comparisons, one-way analysis of variance followed by Dunnett’s multiple comparison testing was performed (GraphPad Prism 6, GraphPad Software, La Jolla, CA, USA). To assess the significance of the impact of biomarker gene expression levels on safety classification, ordinal logistic regression analysis was performed (JMP 12.0.1 statistical software, SAS Institute, Cary, NC, USA). Specifically, ordinal logistic regression analysis was performed to assess the predictability of biomarker gene expression by evaluating the relationship between biomarker gene expression levels and vaccine safety categories. Three safety categories were used: group 1 for SA and HAv, group 2 for poly I:C-HAv combined, and group 3 for the RE. Analysis was performed using the following equation.

$$\text{ln}\left(\frac{p}{1-p}\right)=\beta_{0}+\beta_{1}\times (\text{relative}\;\text{gene}\;\text{expression}\;\text{level})$$

where *p* is the probability of each category, the left side of the equation is the logit value between two categories, and β_0_ and β_1_ are the coefficient values for the equation. To assess the predictability calculated by the derived equation, biomarker gene expression levels were substituted into the derived equation. The appropriate standardized coefficient (β) values for the equation are shown in S1–S3 Tables. The results are indicated as the classification rate for RE or poly I:C combined with HAv. The data represent the standardized coefficient (β) value for poly I:C combined with HAv. The results predicted from hierarchical clustering analyses with Pearson correlation and average linkage were generated using Multiple Experiment Viewer (Mev) software package ver. 4.8.1 (http://mev.tm4.org).

To assess the relationship for the classification results between two inoculation routes, ordinal logistic regression analysis was performed. The average value of each predicted value for each group was used for ordinal logistic regression analysis to obtain corresponding results of each inoculation route for each inoculated vaccine. Three safety categories were used for the SA group, HAv group, AddaVax^™^-HAv combination group, poly I:C-HAv combination group, and RE group. Analysis was performed using the following equation.

$$\text{ln}\left(\frac{p}{1-p}\right)=\beta_{0}+\beta_{1}\times (ip)+\beta_{2}\times (na\;\text{or}\;im\text{)}$$

where *p* is the probability of each category and the left side of the equation is the logit value between two categories. β_0_, β_1_, and β_2_ are the coefficient values in the equation and *ip*, *na*, and *im* indicate the mean substituted values for the gene expression level of the intraperitoneal, intranasal, and intramuscular groups, respectively. When the odds were the same between categories, lines separating the zone were drawn where the logit values were zero and the equation was rearranged to yield the following:

$$\left(nasal\;\text{or}\;im)=\frac{\beta_{0}}{\beta_{2}}-\frac{\beta_{1}}{\beta_{2}}\times (ip\right)$$

## Results

### Body weight and WBC changes in BALB/c mice with adjuvanted, non-adjuvanted HAv, or toxicity reference vaccine (RE)

**Fig 1:**
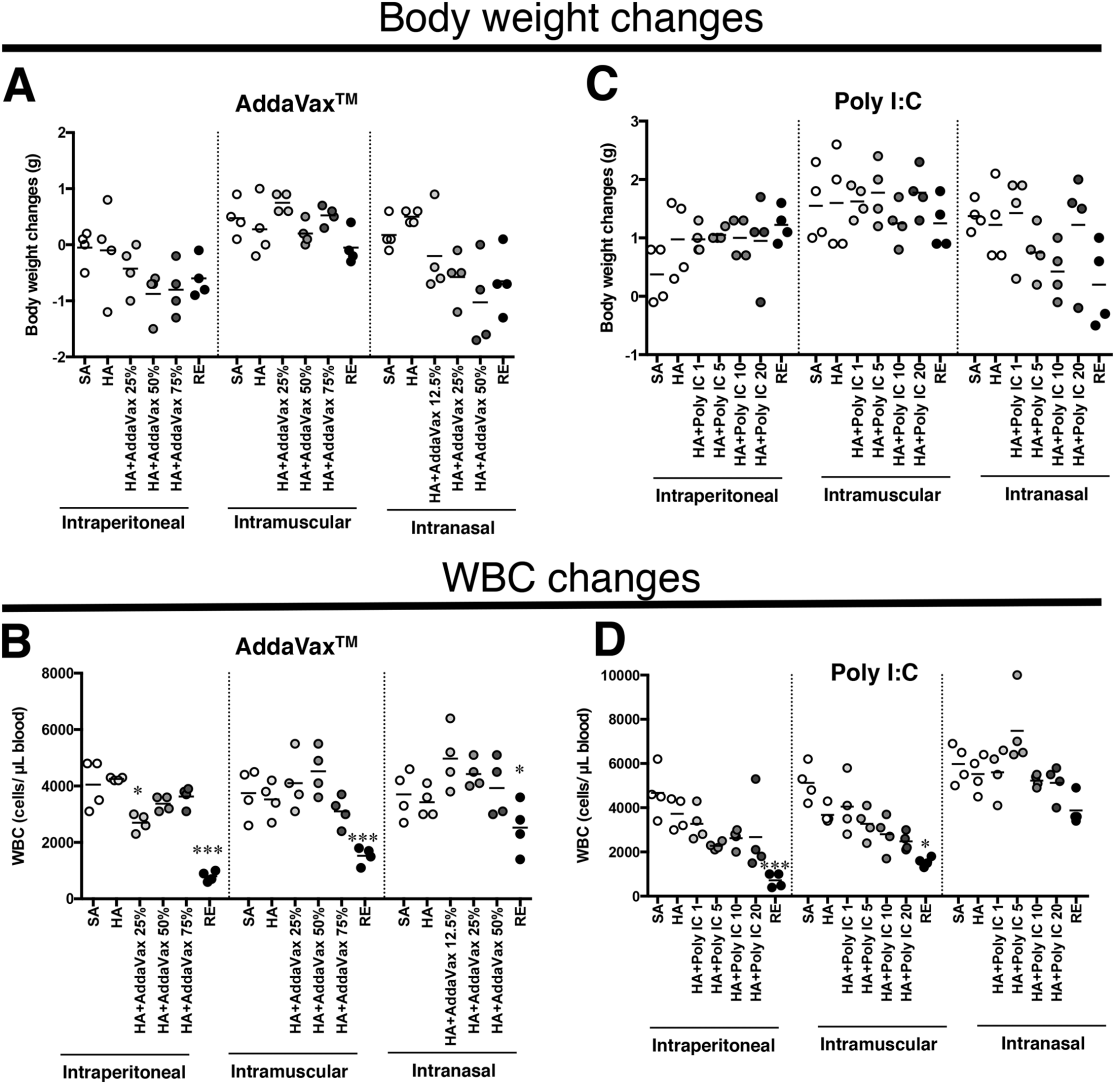
Body weight reduction and white blood cell (WBC) reduction 16 hours after vaccination. (A) and (B) represent the results for the AddaVax^™^-adjuvanted vaccine, and (C) and (D) represent the results for the Poly I:C-adjuvanted vaccine. (A and C) Body weights were measured before and 16 h after vaccination, after which the body weight difference before and after vaccination was calculated for the individual animals. The horizontal lines represent mean body weight changes. (B and D) The numbers of WBCs in the blood were measured at 16 h post-vaccination. The horizontal lines indicate the mean body weight changes. Each plot represents the results for each individual animal. Each group constituted 4 animals. *p < 0.05 and p*** < 0.001 compared with the HA-inoculated mice.

In this study, we prepared two test batches of AddaVax^™^- and poly I:C-adjuvanted vaccines. AddaVax^™^ is a squalene-based oil-in-water emulsion formulation similar to MF59^23^. MF59 has been reported as a safe and potent adjuvant for use with human vaccines, with more than 27 million doses already administered in humans [22]. Therefore, we used AddaVax^™^ as an adjuvant because of its safety and efficacy. Fig 1A and 1B show the results for AddaVax^™^-adjuvanted HAv, while Fig 1C and 1D show the results for poly I:C-adjuvanted HAv. We initially analyzed body weight changes and WBC reduction using the ATT and LTT methods [2], respectively.

Body weight changes and WBC counts in mice were analyzed at 16 h post-vaccination. The vaccine and adjuvant were administered via the intraperitoneal, intramuscular, or intranasal routes. The body weight changes are shown pre- and post-vaccination. Significant changes in body weight were not observed for any concentration of AddaVax^™^-adjuvanted HAv or poly I:C-adjuvanted HAv for the intraperitoneal and intramuscular routes (Fig 1A and 1C). For the intranasal route, body weight was generally decreased when RE, 50% AddaVax^™^, or 10 μg of poly I:C-adjuvanted HAv was administered (Fig 1A and 1C).

AddaVax^™^-adjuvanted HAv did not significantly decrease WBC numbers in the mice by any dosing route (Fig 1B), suggesting that AddaVax^™^ does not have leucopenic toxicity in mice. Consistent with these results, AddaVax^™^ and leucopenic toxicity has not been reported in an animal model or clinical report [21,25]. In contrast, poly I:C-adjuvanted HAv-treated mice showed decreased WBC numbers in a dose-dependent manner except for the intranasal route (Fig 1D). These results correspond to those of previous studies showing that poly I:C has immunotoxicity in animals and humans [26,27].

As reported previously, whole virion inactivated influenza vaccines or live attenuated influenza vaccines cause leucopenic reactions in mice and rabbits [26,27]. Thus, we adopted RE, a whole-virion inactivated influenza vaccine used as a toxicity reference vaccine in LTT in Japan [2]. RE administration significantly reduced WBC counts in mice inoculated by any route (Fig 1B and 1D), except nasal inoculation, as shown in Fig 1D.

### Gene expression analysis of lung tissue from mice inoculated with AddaVax^™^-adjuvanted vaccine, poly I:C-adjuvanted vaccine, HAv, or RE

The expression levels of 18 lung biomarkers, the information for which is shown in Table 2, were analyzed. These genes were selected because their expression levels have been shown to be correlated with body weight loss and WBC levels after influenza vaccination by intraperitoneal injection [23,24]. Therefore, these 18 genes can be used as biomarkers for ATT and LTT. Mouse lungs were collected at 16 h post-vaccination, total RNA was extracted, and QuantiGene Plex (QGP) analysis was conducted according to previous reports [23,24].

**Fig 2:**
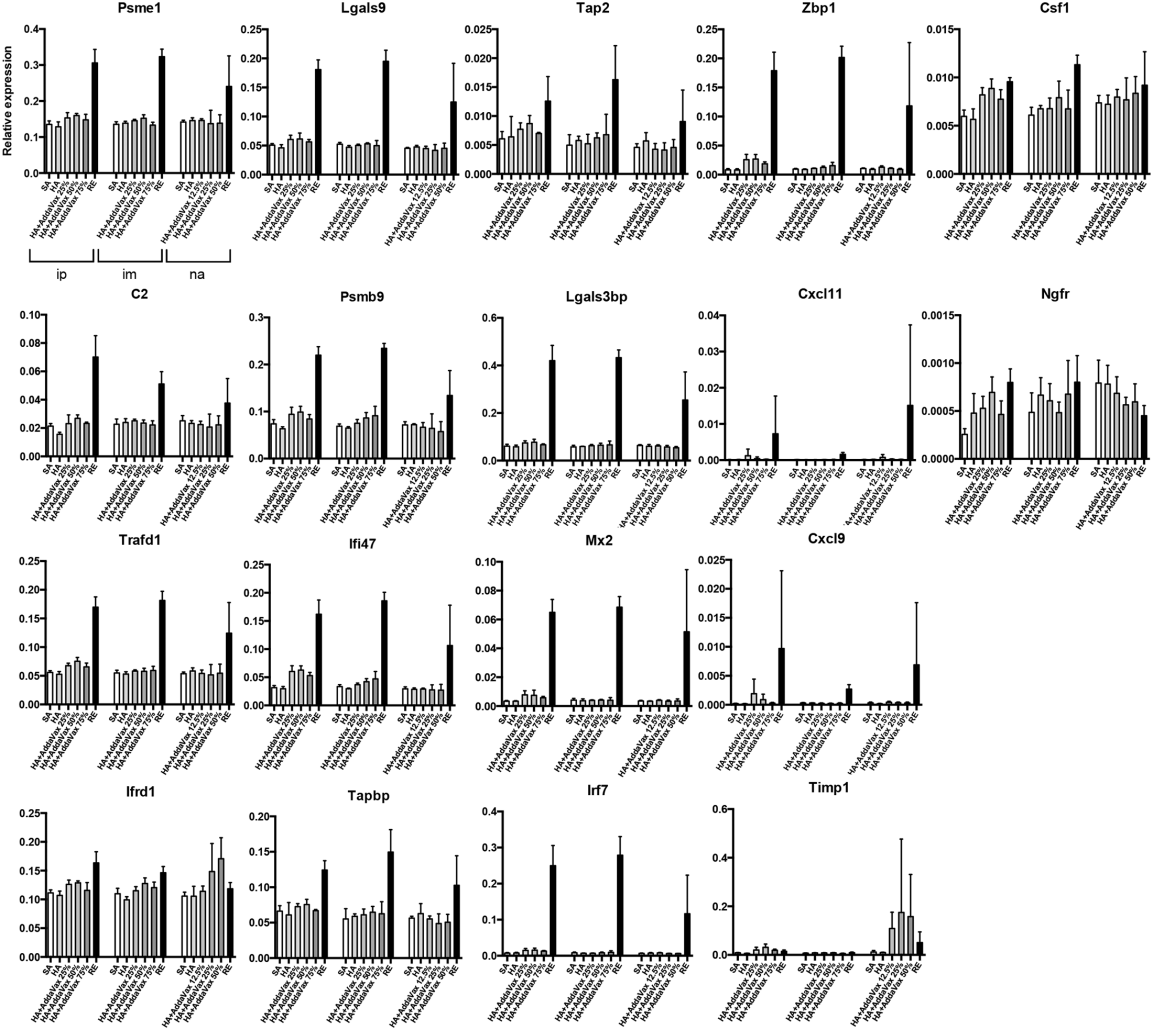
Changes in the expression levels of 18 lung biomarkers 16 hours after AddaVax^™^-adjuvanted vaccination. At 16 h post-vaccination, mouse lungs were collected and total RNA was extracted from them. The total RNA was used for QGP analysis to assess the expression levels of the biomarkers. Their expression levels are shown as relative expression levels normalized against ß-actin. The data represents mean ±SD. In the figure, ip, im, and na indicate intraperitoneal, intramuscular and nasal vaccination routes, respectively. Each group constituted by 4 animals.

**Fig 3:**
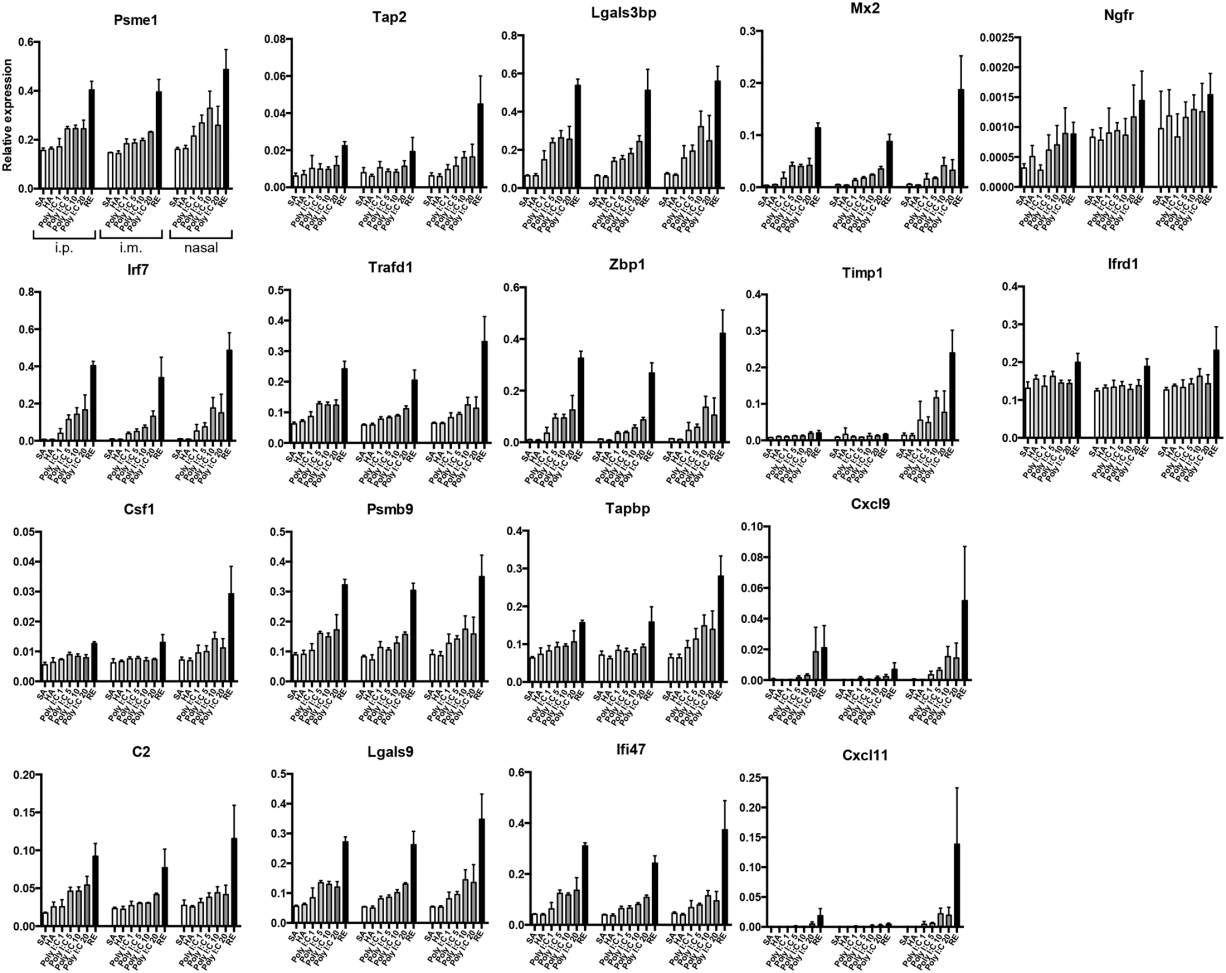
Changes in the expression levels of 18 lung biomarkers 16 hours after Poly I:C-adjuvanted vaccination. At 16 h post-vaccination, mouse lungs were collected and total RNA was extracted from them. The total RNA was used for QGP analysis to assess the expression levels of the biomarkers. Their expression levels are shown as relative expression levels normalized against ß-actin. The data represents mean ±SD. In the figure, ip, im, and na indicate intraperitoneal, intramuscular and nasal vaccination routes, respectively. Each group constituted by 4 animals.

In the AddaVax^™^ batch experiment, except for *Timp1*, *Cxcl9*, and *Cxcl11*, no notable elevations in gene expression levels were observed in the AddaVax^™^-adjuvanted HAv-treated mice (Fig 2). However, in the poly I:C batch study, treatment of mice with the poly I:C AHv combination induced increased gene expression levels in a dose-dependent manner, except for *Csf1* and *Ifrd1* (Fig 3). As described in our previous report, these biomarkers were identified as RE-specific responsive genes [5]. Taking this into consideration with the current results, poly I:C may have RE-like toxicity (Fig 3). As shown in Fig 1D, poly I:C-adjuvanted vaccine-treated mice and RE-treated mice showed decreased WBC counts. These results suggest that the biomarker gene responses indicate adjuvant-induced RE-like immunotoxicity. The RE-like immunotoxicity of poly I:C HAv was suggested by the expression profiles of the 18 biomarkers. Interestingly, the poly I:C HAv treatment combination, when administered intranasally, increased biomarker gene expression levels, but no WBC reductions were observed (Fig 1D), suggesting that the biomarkers can be used to bypass WBC reduction analysis for the intranasal route. S4–S9 Tables show the raw expression data for the 18 genes examined in this study.

### Predictability of the expression levels of the 18 genes for vaccine safety categorization and selection of a highly predictive gene set for vaccine safety classification

To assess the predictability of the expression levels of the 18 genes for vaccine safety categorization, the vaccines and adjuvants were divided into the following 3 groups: group 1 included SA and HAv, group 2 included poly I:C-adjuvanted HAv, and group 3 included RE. We omitted AddaVax^™^-adjuvanted vaccines from the analyses, as they did not elevate biomarker gene expression levels compared with HAv (Fig 2). Therefore, we considered that AddaVax^™^ could be substituted for HAv based on the expression patterns of the 18 biomarkers. Additionally, each vaccination route was individually analyzed. To investigate the predictability of each of the 18 genes for vaccine safety categorization, ordinal logistic regression analysis was performed (S1–S3 Tables). The concept underlying the use of the biomarkers as predictive tools is that if the biomarker gene expression levels are correlated with the degree of RE-like immunotoxicity and immunogenicity, their expression levels may be superior predictable factors for vaccine and adjuvant safety.

The intranasal results indicated that, except for *Trafd1*, *Irf7*, *Cxcl11*, and *Zbp1*, 14 genes showed significant differences (*p* < 0.05); among these genes, *Ifi47* was the most important factor because it showed the highest *ß*_*1*_ value (S1 Table and Methods). Among the 18 genes, *Ngfr* and *Ifrd1* showed low logit r^2^ values of less than 0.5 in the results of the whole-model test (S1 Table). Therefore, these two genes are not suitable as predictors of vaccine and adjuvant safety. The predictive biomarker gene set for vaccine and adjuvant safety was extracted using the following criteria: the gene set has a *p* value of <0.05 and logit *r*^*2*^ value ≥0.5 in the whole-model test results. As a result, 12 genes (i.e., *Psme1*, *Timp1*, *Tap2*, *C2*, *Psmb9*, *Cxcl9*, *Csf1*, *Lgals9*, *Lgals3bp*, *Mx2*, *Ifi47*, and *Tapbp*) were selected as the vaccine and adjuvant safety prediction classification gene set for the intranasal route.

Using the same process as used for the intranasal route, we assessed the same 18 genes for their predictive potential by ordinal logistic regression analyses followed by gene selection for the intramuscular and intraperitoneal routes. For the intramuscular route (except for *Timp1*, *Cxcl11*, and *Ngfr*), 15 genes showed significant differences (*p* < 0.05) in each of the three groups and among groups, with *Psme1* as the most important factor because its value *ß*_*1*_ value was the highest (S2 Table). Among the 18 genes, *Timp1*, *Tap2*, *Cxcl11*, *Psmb9*, *Cxcl9*, *Csf1*, *Ngfr*, and *Irfd1*) showed low logit *r*^*2*^ values in the whole-model test (S2 Table). Therefore, 10 genes (*i*.*e*., *Psme1*, *C2*, *Trafd1*, *Irf7*, *Lgals9*, *Lgals3bp*, *Zbp1*, *Mx2*, and *Ifi47*) were selected as the gene set for predicting vaccine and adjuvant safety classification for the intramuscular route according to the criteria described above for intranasal inoculation. For the intraperitoneal route, except for 8 genes (i.e., *Psme1*, *C2*, *Trafd1*, *Psmb9*, *Cxcl9*, *Ngfr*, *Lgals9*, *Lgals3bp*, and *Mx2)*, 10 genes showed significant differences (*p* < 0.05) in each of the three groups, among which *Irfd1* was the most important factor because its *ß*_*1*_ value was the highest (S3 Table). Among the 18 genes, *Timp1*, *Tap2*, *Cxcl11*, *Cxcl9*, *Ngfr*, and *Irfd1* showed low logit *r*^*2*^ values for the whole-model test results (S3 Table). Therefore, 5 genes, *Irf7*, *Csf1*, *Zbp1*, *Ifi47*, and *Tapbp*, were selected as the gene set for predicting vaccine and adjuvant safety classification for the intraperitoneal route. However, only 5 biomarker genes were selected to evaluate the intraperitoneal route, which is small to achieve accurate evaluation. Therefore, in this evaluation system, nasal administration and intramuscular administration were considered more robust than intraperitoneal administration.

**Fig 4:**
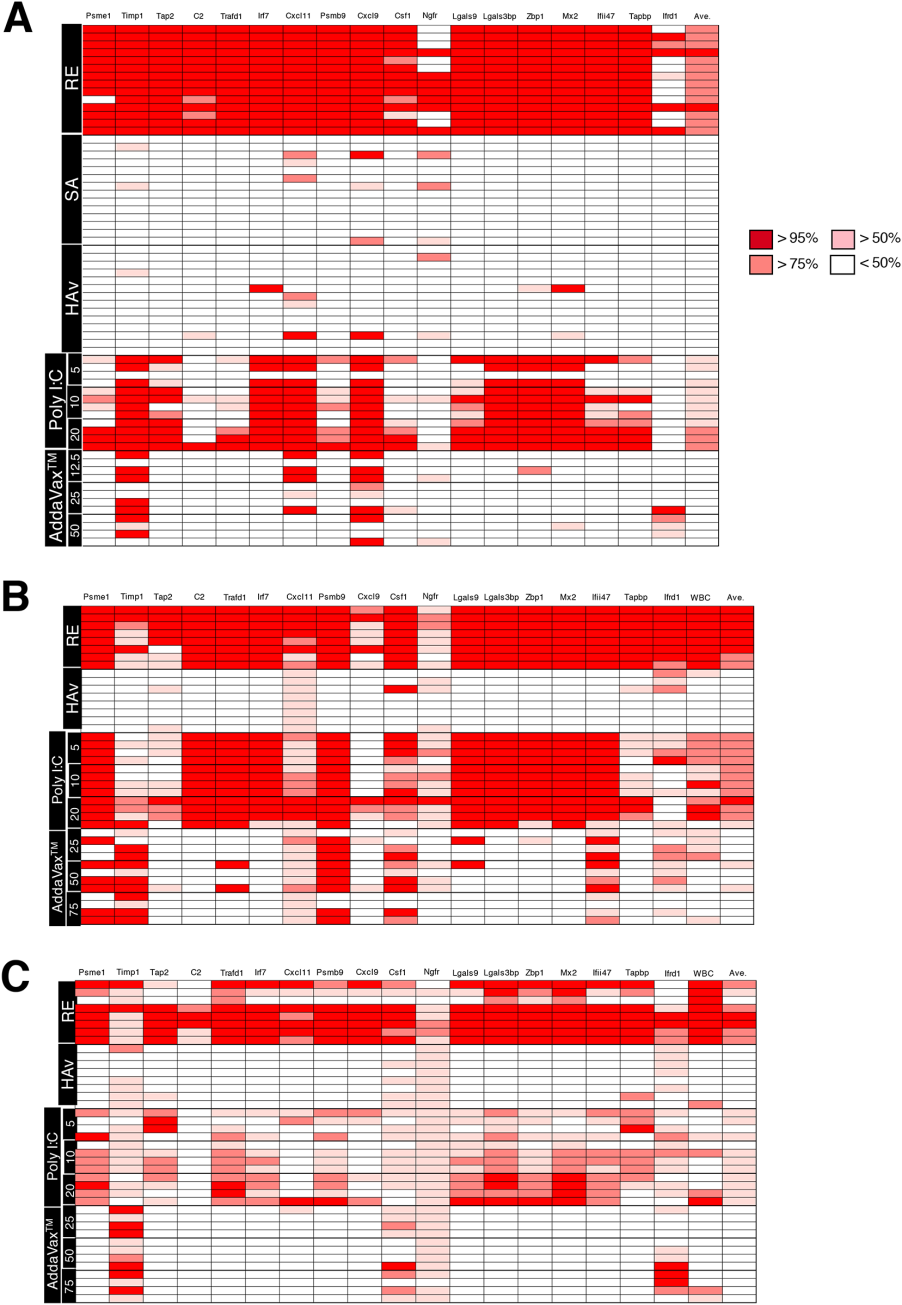
Predicted profiles of the 18 biomarkers for vaccine safety categorization. In the heat map, each row is a type of vaccine and each column is a biomarker gene. The heat map is coloured by the probability classification, as defined by RE-like toxicity as an outcome (red rectangles), while white rectangles are defined as lacking RE-like toxicity. The predicted value was calculated from the biomarker gene expression levels via the equation from the ordinal logistic regression analysis. Each row indicates the result for an individual animal. A, B and C represent intranasal, intramuscular, and intraperitoneal vaccination routes, respectively.

Actual predictions for vaccine classification from the gene expression profiles were performed using the regression equation for poly I:C-adjuvanted HAv of *ß*_*0*_. This was because the criteria for poly I:C-adjuvanted AHv are more severe for toxicity assessment compared to RE and because biomarker gene expression levels for poly I:C adjuvanted-HAv were lower than those of RE. *ß*_*0*_ and *ß*_*1*_ were substituted with the appropriate values obtained from the results of ordinal logistic regression analyses (S1–S3 Tables). These analyses were only performed on the final selected gene set as described above. Next, each biomarker gene expression level was substituted into the regression equation and the probability of the gene being classified as having RE-like immunotoxicity and immunogenicity was calculated. These calculations were performed for each animal (Fig 4A–4C), where each well represents an individual animal and the results for the 18 individual genes. The depth of red color shows the probability of having RE-like toxicity, which was introduced as the actual value of biomarker gene expression and the logistic regression equation. As a result, the prediction value clearly separated RE and HAv (Fig 4A–4C). Like RE, the poly I:C-HAv combination was separated from HAv, but to a lesser extent than from RE. For the AddaVax^™^-adjuvanted HAv, almost all the genes indicated were classified into HAv (Fig 4A–4C). The average predicted values for the 18 genes were calculated in individual mice. The threshold of classification was set to 50% probability classified into RE. For the intranasal route, the average of the predicted values indicated that all animals inoculated with RE or 10 or 20 μg poly I:C-adjuvanted HAv were classified as having RE-like vaccine immunotoxicity (Fig 4A). In contrast, AddaVax^™^-adjuvanted HAv-treated animals were classified as having non-RE-like immunotoxicity. Using the same approach as used for the intranasal route, the intramuscular and intraperitoneal routes were assessed. As a result, nearly all predicted values showed high diagnostic accuracy regardless of whether the vaccine showed RE-like immunotoxicity (Fig 4B and 4C). Among the 18 genes, the omitted genes described above showed low diagnostic accuracy (e.g., *Ngfr* and *Cxcl11* in the intranasal route) (Fig 4A).

Clustering analyses were performed using the predicted values (S1A–S1C Fig). The results indicated that RE and high concentration poly I:C-adjuvanted vaccines were distantly clustered to HAv and SA in any vaccination route. For the intranasal route, AddaVax^™^-adjuvanted HAv sub-clustered between HAv and low-concentration poly I:C (S1C Fig).

These results suggest that the logistic regression equation-introduced predicted value has high predictability for vaccine and adjuvant safety classification.

### Comprehensive classification of vaccination routes

**Fig 5:**
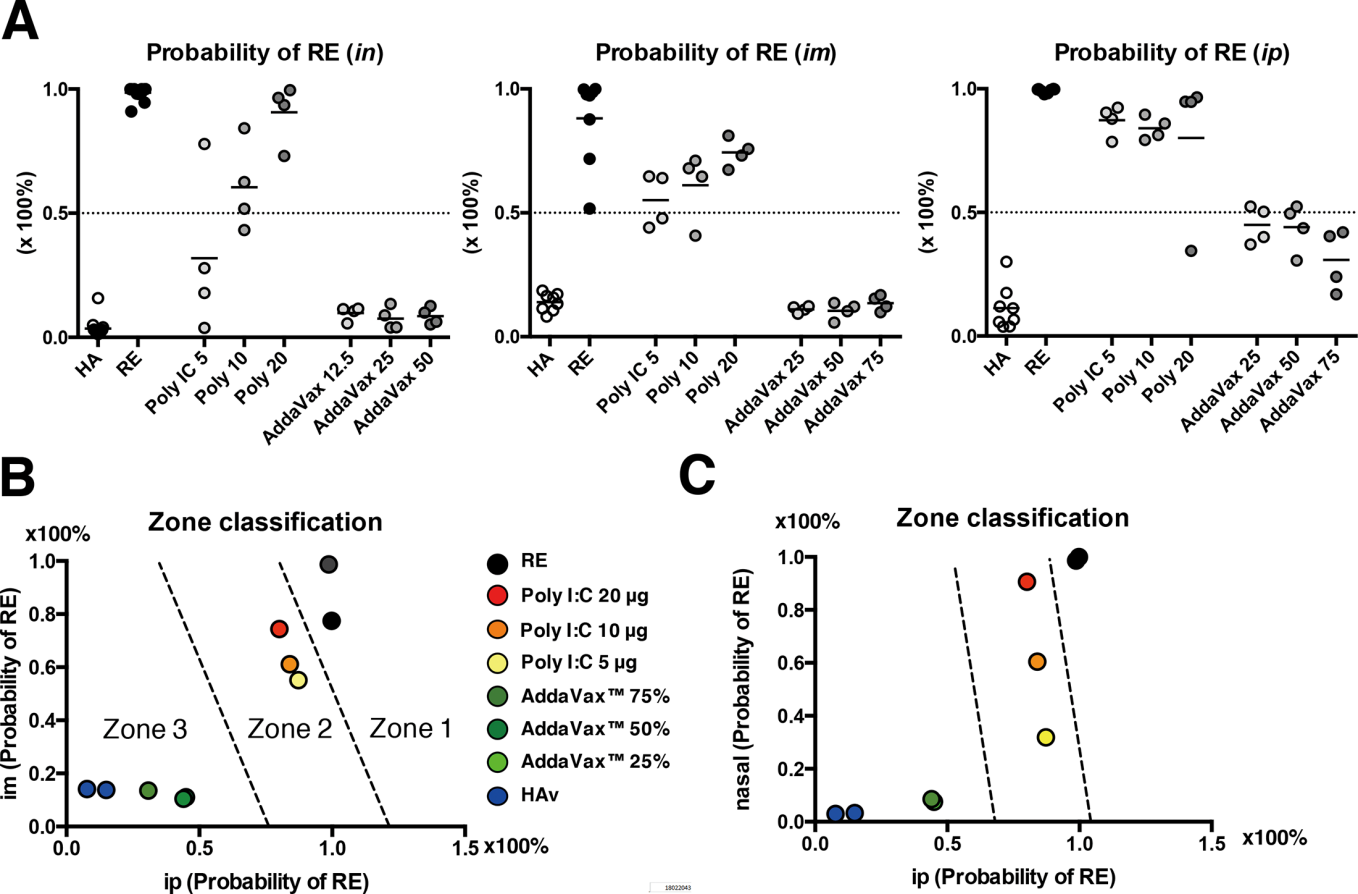
Comprehensive evaluation of influenza vaccine safety by zone classification. (A) The classification analysis using the probability value classified for RE. The probability indicated is the probability classified for RE by biomarker gene expression as served by the regression equation and the biomarker gene expression values. The probability is represented by 0 to 100%; thus, the threshold can be set to 50% probability. The results represent intranasal (*in*), intramuscular (*im*) and intraperitoneal (*ip*) vaccination routes. Each group constituted by 4 animals. Representative zone classification for RE-like toxicity risk assessment showing the intraperitoneal *vs* intramuscular vaccination route (B), and the intraperitoneal *vs* intranasal vaccination route (C). Each plot represents the mean value for each vaccine-treated group (4 animals/group). The broken lines separating the zones are drawn where the logit values were zero; the detailed method is described in Methods.

To summarize the vaccine and adjuvant safety classifications provided by the gene sets, the average predicted values were employed. The results showed that all dosing routes were clearly classified in the analysis (Fig 5A). Particularly, RE-treated and poly I:C-adjuvanted HAv-treated animals were classified into the appropriate zone indicative of having RE-like immunotoxicity (≥50% of probability classified into RE). In contrast, AddaVax^™^-adjuvanted HAv-treated animals were classified into the zone indicative of having non-RE-like immunotoxicity (<50% of probability classified into RE), as were the SA- and HAv-treated animals. To assess the relationships among the three vaccination route-dependent predictive values, ordinal logistic regression analysis was performed (S10 Table), using the average predicted value for each group shown in Fig 5A.

From the results of the ordinal logistic regression analysis, two separation lines were drawn in each correlation figure for which the odds were unity between HAv and poly I:C combined with HAv and between poly I:C combined with HAv and RE. We assigned the line-separated zones as having non-RE-like toxicity (zone 3), having RE-like toxicity (zone 2), and almost having RE toxicity (zone 1). The results indicate that RE-treated animals were in zone 1 and poly I:C HAv combination-treated animals were in zone 2, while for both intraperitoneal *vs* intranasal or intramuscular injection, the others were in zone 3 (Fig 5B and 5C). Each vaccination route correlated with the other vaccination routes, suggesting that our zone classification analysis is a powerful toxicity assessment tool for different vaccination routes.

## Discussion

To assess vaccine safety and quality in this study, we established a vaccine safety profiling and prediction system using biomarker gene expression profiles identified by ordinal logistic regression analysis as the best predictors. Combined evaluation of the two vaccination routes (i.e., intramuscular *vs* intraperitoneal, Fig 5B and nasal *vs* intraperitoneal, Fig 5C, produced clearly separated zone classifications) confirmed the reliability of zone classification, with each zone separated by a border line along which the logit value was zero, thus defining zones 1–3 (Fig 5B and 5C). The safety categories were well-separated by these zones. This good correlation for the two vaccination routes indicates that our zoning system can be used for safety assessment of influenza vaccines administered intranasally, intramuscularly, and intraperitoneally.

RE was used as a toxicity control to design the evaluation system; however, other vaccine or adjuvant types can be set as toxicity reference formulations. The results obtained will differ depending on the type of toxicity control. However, it is possible to evaluate the desired toxicity types by appropriately selecting the toxicity control formulation to be evaluated. Here, poly I:C was used as the toxic adjuvant; therefore, from the toxicity evaluation based on RE as the toxicity control, poly I:C showed similar toxicity to RE (Figs 3 and 5A). This finding is consistent with those of previous reports in which poly I:C was shown to have blood toxicity (leucopenia) as observed for RE [27]. In contrast, RE-based toxicity has not been detected with AddaVax^™^ adjuvant, which has relatively fewer reported side effects [21,22], whereas we predict that poly I:C has RE-like toxicity both towards WBCs and general toxicity. Therefore, toxicity evaluations based on RE are very useful for evaluating inactivated influenza vaccines and adjuvanted vaccines. Here, the influenza vaccine was selected as the formulation. However, the logistic regression-based prediction system for toxicity control can also be applied to safety assessment of vaccines against other pathogens, provided that an adequate toxicity control is available. Further improvements of the evaluation system, such as the production of cytokines, which are directly involved in inflammation, are needed.

Historically, the safety of a vaccine was assessed by weight loss and WBC reduction [26,27–30]. The present study showed that poly I:C- and AddaVaxTM-combined HA reduced WBC compared to in SA-treated mice (Fig 1B and 1C). The time point of WBC assessment was set based on the LTT. The LTT detect the influenza vaccine-induced leukopenic reactions. In previous studies, whole virion-inactivated influenza vaccine-induced WBC reduction peaked at 16 h after vaccination, and then gradually recovered by 24 h after vaccination [28,29]. Thus, leukopenic reactions may be transient in AddaVax- and Poly I:C-treated animals. However, in recent years, attempts have been made to develop a toxicity evaluation system using biomarker gene expression with genomics technologies [4,7,31]. These methods have already been adopted for safety evaluation of synthetic medicines and industrial chemicals. However, most reports are limited to biomarker identification. Few studies have examined how to evaluate toxicity and safety based on changes in biomarker expression patterns. The goal of pharmaceutical and biological product safety assessments is to obtain robust data regarding safety and toxicity. Hence, our safety assessment system was constructed using statistical and quantitative methods derived from biomarker gene expression profiles. The logistic regression analysis method used in this study has been used for the prognosis and diagnosis of lifestyle-related diseases such as diabetes and hyperlipidemia [32–34]. We hypothesized that such a predictive diagnostic method could be applied for safety prediction in vaccines. Particularly, by dividing the type of vaccine for each toxicity level, the test sample can be divided into each type of toxicity using a regression equation. In the current study, the reference vaccines were classified into three types: SA, HAv, and AddaVax^™^ (combined with HAv for safety), poly I:C-HAv for adjuvant toxicity, and RE for vaccine toxicity. For example, the safety of a test sample can be assessed initially by evaluating its toxicity type classification. If it is classified as an RE type, the test specimen is likely to have similar toxicity to RE. This type of practical safety assessment can be applied not only to vaccines and adjuvants, but also to he safety evaluation of synthetic medicines and industrial chemicals.

In evaluating vaccine safety, the influence of the administration route is very important, as it is likely to affect toxicity and because the immune response elicited will differ according to the vaccination route [13–15]. Using traditional vaccine safety assessment methods typified by ATT and LTT, the vaccine is administered intraperitoneally [2,26,27,30], as the purpose is to evaluate general systemic toxicity [26]. However, to capture more specific and broad toxicity information, evaluation by nasal or intramuscular administration is preferred [35]. Hence, in addition to intraperitoneal administration, we evaluated nasal and intramuscular administration, which are thought to have a large influence on the inoculation site. Interestingly, the expression profile of a biomarker gene was equivalent across all administration routes (Figs 2 and 3). In biomarker gene expression analyses, we used RNA from the lungs for all administration routes. This suggests that the lungs play an important role in the immune response against influenza vaccines. Intriguingly, we observed nearly the same gene expression pattern in lung distant from the inoculation site by any administration route (Figs 2 and 3). Thus, our findings indicate that the safety assessment markers behaved independently of the administration route and suggest the importance of the lungs when evaluating vaccine safety. However, this finding is limited to the influenza vaccine; therefore, this method should be tested for other pathogen vaccines in future studies.

The biomarker gene sets evaluated in this study were type-1 interferon (IFN)-related or IFN-inducible genes as typified by *Irf7*, *Lgals3bp*, and *Lgals9* [36–38], suggesting that one of the biological effects of RE detected by the biomarkers involves type-1 IFN-related signals. In addition, *Ifi47*, *Mx2*, *Tap2*, and *Psmb9* induction by type-1 IFN has been reported [39]. Influenza virus, which can induce type-I IFNs, is known to be involved in leucopenic toxicity, suggesting that these genes are partially involved in influenza vaccine-induced leucopenic toxicity [40]. Furthermore, excessive type-1 IFN signaling in response to acute influenza infection results in uncontrolled inflammation and TNF-related apoptosis-inducing ligand–death receptor 5-mediated epithelial cell death [41].

The genes identified in the present study are involved in the effectiveness of influenza vaccines. For example, *Ifi47*, a commonly selected predictive biomarker gene in all three vaccination route types, is a member of the IFN-induced GTPase family, which play important roles in the responses to various pathogens [42]. Additionally, IFNγ-inducible *Psme1* is a component of immunoproteasomes, which are involved in generating MHC-I-presented peptides [43]. Based on these previous studies, we examined whether biomarkers can be used to evaluate not only toxic effects, but also reactogenicity and effectiveness. In fact, the effectiveness of a vaccine cannot often be clearly separated from its toxic effects. For example, as described above, type-1 IFN has antiviral effects and causes uncontrolled inflammation.

The functions of most biomarker genes are considered to have been preserved in mice and humans [36–41]. To extrapolate presently used vaccine evaluation systems to human reactions, it is necessary to conduct evaluate human samples. As described above, the biomarker genes include type-1 IFN signal- and antigen presentation-related genes. These biomarker genes can be applied in vaccine evaluation not only for lung samples, but also for blood samples. Taken together, to make this evaluation system practical, verification using human peripheral blood mononuclear cells (PBMC) or human peripheral blood lymphocytes humanized models is necessary to extrapolate the evaluation to humans.

In conclusion, we established a novel, comprehensive evaluation system for vaccines and adjuvants. The evaluation is based on a lung biomarker gene set and statistical methodology, enabling rapid, objective, and comprehensive evaluation. We also examined the influence of the vaccination route, which has not been widely considered in conventional evaluation systems, and found that the proposed system was only minimally affected by the administration route. Therefore, our evaluation system is useful not only in the alternative test method for ATT and LTT, but also during drug development stages such as preclinical testing. Predictive factors such as gene expression and the logistic regression analysis prediction system and zone classification for the two administration routes are novel and can be applied to vaccines other than the influenza vaccine. Moreover, this system can be applied to vaccines as well as safety evaluations of synthetic medicines and industrial chemicals. Additional verification of other adjuvants and vaccine preparations is needed to support the usefulness of our evaluation system. This technology can be applied in animal experiments as well as cell culture-based evaluation systems. These findings are useful for developing safe vaccine adjuvants and improving the accuracy of quality control tests for biological products, as typified by ATT and LTT.

## Supporting information

S1 Fig Clustered profile of the predicted outcome of RE-like toxicity. In the heat map, each row is a type of vaccine and each column is a biomarker gene. The heat map is colored by the probability of having RE-like toxicity outcome; blue colors indicate more distinction from RE property, and red colors indicate more closeness to RE property. Vaccines are grouped into clusters of similar predicted profiles. The result shows cases of (a) intranasal, (b) intramuscular, and (c) intraperitoneal vaccination.
(TIFF)

S1 Table Ordinal logistic regression analysis of biomarkers in the nasal inoculation group. (DOCX)

S2 Table Ordinal logistic regression analysis of biomarkers in the intramuscular inoculation group. (DOCX)

S3 Table Ordinal logistic regression analysis of biomarkers in the intraperitoneal inoculation group. (DOCX)

S4 Table The biomarkers expression profiles in Advax group. Data are presented as the mean ± S.D.
(DOCX)

S5 Table The biomarkers expression profiles in Advax group. Data are presented as the mean ± S.D.
(DOCX)

S6 Table The biomarkers expression profiles in Advax group. Data are presented as the mean ± S.D.
(DOCX)

S7 Table The biomarkers expression profiles in Poly I:C group. Data are presented as the mean ± S.D.
(DOCX)

S8 Table The biomarkers expression profiles in Poly I:C group. Data are presented as the mean ± S.D.
(DOCX)

S9 Table The biomarkers expression profiles in Poly I:C group. Data are presented as the mean ± S.D.
(DOCX)

S10 Table Ordinal logistic regression analysis for the average of the predicted outcomes in *i*.*p*. and *i*.*m*. or *i*.*p*. and nasal administered group. (DOCX)

## References

[1] Public Health Service Act. 1944 (as amended). 42 U.S.C. 6A. http://www.FDA.gov/RegulatoryInformation/Legislation/ucm148717.htm.

[2] National Institute of Infectious Diseases. Minimum Requirements for Biological Products. Japan: National Institute of Infectious Diseases, General Tests, 272–336 and Influenza Vaccine. 2006:10–13.

[3] Hamaguchi I, Imai J, Momose H, Kawamura M, Mizukami T, Kato H, et al Two vaccine toxicity-related genes Agp and Hpx could prove useful for pertussis vaccine safety control. Vaccine. 2007;25: 3355–3364. doi: 10.1016/j.vaccine.2006.12.059 17280746 10.1016/j.vaccine.2006.12.059

[4] Hamaguchi I, Imai J, Momose H, Kawamura M, Mizukami T, Naito S, et al Application of quantitative gene expression analysis for pertussis vaccine safety control. Vaccine. 2008;26: 4686–4696. doi: 10.1016/j.vaccine.2008.06.086 18619509 10.1016/j.vaccine.2008.06.086

[5] Poland GA, Borrud A, Jacobson RM, McDermott K, Wollan PC, Brakke D, et al Determination of deltoid fat pad thickness: implications for needle length in adult immunization. JAMA. 1997;277:1709–1711. 9169899

[6] Shaw FE Jr, Guess HA, Roets JM, Mohr FE, Coleman PJ, Mandel EJ, et al Effect of anatomic site, age and smoking on the immune response to hepatitis B vaccination. Vaccine. 1989;7: 425–430. 2530717 10.1016/0264-410x(89)90157-6

[7] Groswasser J, Kahn A, Bouche B, Hanquinet S, Perlmuter N, Hessel L. Needle length and injection technique for efficient intramuscular vaccine delivery in infants and children evaluated through an ultrasonographic determination of subcutaneous and muscle layer thickness. Pediatrics. 1997;100: 400–403. 9282716 10.1542/peds.100.3.400

[8] Ghimire TR. The mechanisms of action of vaccines containing aluminum adjuvants: an *in vitro vs in vivo* paradigm. Springerplus. 2015;4: 181 doi: 10.1186/s40064-015-0972-0 25932368 10.1186/s40064-015-0972-0 PMC4406982

[9] Durando P, Icardi G, Ansaldi F. MF59-adjuvanted vaccine: a safe and useful tool to enhance and broaden protection against seasonal influenza viruses in subjects at risk. Expert Opin Biol Ther. 2010;10: 639–651. doi: 10.1517/14712591003724662 20218923 10.1517/14712591003724662

[10] Ortbals DW, Liebhaber H. Comparison of immunogenicity of a whole virion and a subunit influenza vaccine in adults. J Clin Microbiol. 1978;8: 431–434. 721947 10.1128/jcm.8.4.431-434.1978 PMC275266

[11] Soema PC, Kompier R, Amorij JP, Kersten GF. Current and next generation influenza vaccines: formulation and production strategies. Eur J Pharm Biopharm. 2015;94: 251–263. doi: 10.1016/j.ejpb.2015.05.023 26047796 10.1016/j.ejpb.2015.05.023

[12] Cook IF. Evidence based route of administration of vaccines. Hum Vaccin. 2008;4: 67–73. 17881890 10.4161/hv.4.1.4747

[13] Lago-Deibe FI, Martín-Miguel MV, Velicia-Peñas C, Gómez-Serranillos IR, Fontanillo-Fontanillo M. The safety and efficacy of the tetanus vaccine intramuscularly versus subcutaneously in anticoagulated patients: a randomized clinical trial. BMC Fam Pract. 2014;15: 147 doi: 10.1186/1471-2296-15-147 25168768 10.1186/1471-2296-15-147 PMC4158096

[14] Diez-Domingo J, Weinke T, Garcia de Lomas J, Meyer CU, Bertrand I, Eymin C, et al Comparison of intramuscular and subcutaneous administration of a herpes zoster live-attenuated vaccine in adults aged ≥50 years: a randomised non-inferiority clinical trial. Vaccine. 2015;33: 789–795. doi: 10.1016/j.vaccine.2014.12.024 25555381 10.1016/j.vaccine.2014.12.024

[15] Delafuente JC, Davis JA, Meuleman JR, Jones RA. Influenza vaccination and warfarin anticoagulation: a comparison of subcutaneous and intramuscular routes of administration in elderly men. Pharmacotherapy. 1998;18: 631–636. 9620115

[16] Marichal T, Ohata K, Bedoret D, Mesnil C, Sabatel C, Kobiyama K, et al DNA released from dying host cells mediates aluminum adjuvant activity. Nat Med. 2011;17: 996–1002. doi: 10.1038/nm.2403 21765404 10.1038/nm.2403

[17] Mizukami T, Imai J, Hamaguchi I, Kawamura M, Momose H, Naito S, et al Application of DNA microarray technology to influenza A/Vietnam/1194/2004 (H5N1) vaccine safety evaluation. Vaccine. 2008;24: 2270–2283. 10.1016/j.vaccine.2008.02.031 18374459

[18] Sasaki E, Kuramitsu M, Momose H, Kobiyama K, Aoshi T, Yamada H. et al A novel vaccinological evaluation of intranasal vaccine and adjuvant safety for preclinical tests. Vaccine, 2017;35: 821–830. doi: 10.1016/j.vaccine.2016.12.036 28063707 10.1016/j.vaccine.2016.12.036

[19] Robinson RA, DeVita VT, Levy HB, Baron S, Hubbard SP, Levine AS. A phase I-II trial of multiple-dose polyriboinosic-polyribocytidylic acid in patients with leukemia or solid tumors. J Natl Cancer Inst. 1976;57: 599–602. 978771 10.1093/jnci/57.3.599

[20] Ichinohe T, Watanabe I, Ito S, Fujii H, Moriyama M, Tamura S, et al Synthetic double-stranded RNA poly(I:C) combined with mucosal vaccine protects against influenza virus infection. J Virol. 2005;79: 2910–2919. doi: 10.1128/JVI.79.5.2910-2919.2005 15709010 10.1128/JVI.79.5.2910-2919.2005 PMC548446

[21] O'Hagan DT, Ott GS, De Gregorio E, Seubert A. The mechanism of action of MF59—an innately attractive adjuvant formulation. Vaccine, 2012;30: 4341–4348. doi: 10.1016/j.vaccine.2011.09.061 22682289 10.1016/j.vaccine.2011.09.061

[22] Schultze V, D'Agosto V, Wack A, Novicki D, Zorn J, Hennig R. Safety of MF59 adjuvant. Vaccine. 2008;26: 3209–3222. doi: 10.1016/j.vaccine.2008.03.093 18462843 10.1016/j.vaccine.2008.03.093

[23] Mizukami T, Momose H, Kuramitsu M, Takizawa K, Araki K, Furuhata K, et al System vaccinology for the evaluation of influenza vaccine safety by multiplex gene detection of novel biomarkers in a preclinical study and batch release test. PLoS ONE. 2017;9: e101835 doi: 10.1371/journal.pone.0101835 25010690 10.1371/journal.pone.0101835 PMC4092028

[24] Momose H, Mizukami T, Kuramitsu M, Takizawa K, Masumi A, Araki K, et al Establishment of a new quality control and vaccine safety test for influenza vaccines and adjuvants using gene expression profiling. PLoS ONE. 2015;10: e0124392 doi: 10.1371/journal.pone.0124392 25909814 10.1371/journal.pone.0124392 PMC4409070

[25] Petrovsky N, Cooper PD. AddaVax^™^, a novel microcrystalline polysaccharide particle engineered from delta inulin, provides robust adjuvant potency together with tolerability and safety. Vaccine. 2015;33: 5920–1926. doi: 10.1016/j.vaccine.2015.09.030 26407920 10.1016/j.vaccine.2015.09.030 PMC4639457

[26] Absher M, Stinebring WR. Toxic properties of a synthetic double-stranded RNA. Endotoxin-like properties of poly I. Poly C, an interferon stimulator. Nature. 1969;5207: 715–717. 10.1038/223715a0 5805520

[27] Philips FS, Fleisher M, Hamilton LD, Schwartz MK, Sternberg SS. Polyinosinic-polycytidylic acid toxicity in: Beers RF, Braun W. (Eds.), *Biological Effects of Polynucleotides*, Springer Verlag, New York 1971: 45–54.

[28] Kurokawa M, Ishida S, Goto N, Kuratsuka K. A new method for biological assay of endotoxin using change in peripheral leukocyte population in mice as a response. Jpn J Med Sci Biol. 1974;27: 173–189. 4546709 10.7883/yoken1952.27.173

[29] Kurokawa M, Ishida S, Asakawa S, Iwasa S, Goto N. Toxicities of influenza vaccine: peripheral leukocytic response to live and inactivated influenza viruses in mice. Jpn J Med Sci Biol. 1975;28: 37–52. 1160196 10.7883/yoken1952.28.37

[30] Mizukami T, Masumi A, Momose H, Kuramitsu M, Takizawa K, Naito S, et al. An improved abnormal toxicity test by using reference vaccine-specific body weight curves and histopathological data for monitoring vaccine quality and safety in Japan. Biologicals. 2009;37: 8–17. doi: 10.1016/j.biologicals.2008.07.007 18805705 10.1016/j.biologicals.2008.07.007

[31] Momose H, Imai J, Hamaguchi I, Kawamura M, Mizukami T, Naito S, et al Induction of indistinguishable gene expression patterns in rats by Vero cell-derived and mouse brain-derived Japanese encephalitis vaccines. Jpn J Infect Dis. 2010;63: 25–30. 20093758

[32] Neter J, Wasserman W. Multiple regression In: Applied Linear Statistical Models. Homewood, Ill: Irwin; 1974: 214–272.

[33] Wilson PW, D'Agostino RB, Levy D, Belanger AM, Silbershatz H, Kannel WB. Prediction of coronary heart disease using risk factor categories. Circulation. 1998;97: 1837–1847. 9603539 10.1161/01.cir.97.18.1837

[34] Ridker PM, Glynn RJ, Hennekens CH. *C*-reactive protein adds to the predictive value of total and HDL cholesterol in determining risk of first myocardial infarction. Circulation. 1998;97: 2007–2011. 9610529 10.1161/01.cir.97.20.2007

[35] Goldenthal KL, Cavagnaro JA, Alving CR, Vogel FR. Safety evaluation of vaccine adjuvants. National cooperative vaccine development working group. AIDS Res Hum Retrovirus. 1993;9: S45–S49.

[36] Schoggins JW, Wilson SJ, Panis M, Murphy MY, Jones CT, Bieniasz P, et al A diverse range of gene products are effectors of the type I interferon antiviral response. Nature. 2011;472: 481–485. doi: 10.1038/nature09907 21478870 10.1038/nature09907 PMC3409588

[37] A dler M, Tavalai N, Muller R, Stamminger T. Human cytomegalovirus immediate-early gene expression is restricted by the nuclear domain 10 component Sp100. J Gen Virol. 2011;92: 1532–1538. doi: 10.1099/vir.0.030981-0 21471311 10.1099/vir.0.030981-0

[38] Satoh T, Kato H, Kumagai Y, Yoneyama M, Sato S, Matsushita K, et al LGP2 is a positive regulator of RIG-I- and MDA5-mediated antiviral responses. Proc Natl Acad Sci USA. 2010;107: 1512–1517. doi: 10.1073/pnas.0912986107 20080593 10.1073/pnas.0912986107 PMC2824407

[39] Wei L, Sandbulte MR, Thomas PG, Webby RJ, Homayouni R, Pfeffer LM. NFkappaB negatively regulates interferon-induced gene expression and anti-influenza activity. J Biol Chem. 2006;281: 11678–11684. doi: 10.1074/jbc.M513286200 16517601 10.1074/jbc.M513286200 PMC1457055

[40] Ato M, Takahashi Y, Fujii H, Hashimoto S, Kaji T, Itamura S, et al Influenza A whole virion vaccine induces a rapid reduction of peripheral blood leukocytes via interferon-α-dependent apoptosis. Vaccine, 2013;31: 2184–2190. doi: 10.1016/j.vaccine.2013.02.016 23434386 10.1016/j.vaccine.2013.02.016

[41] Bucasas KL, Franco LM, Shaw CA, Bray MS, Wells JM, Niño D, et al Early patterns of gene expression correlate with the humoral immune response to influenza vaccination in humans. J Infect Dis. 2011;203: 921–929. doi: 10.1093/infdis/jiq156 21357945 10.1093/infdis/jiq156 PMC3068032

[42] Carlow DA, Teh SJ, Teh HS. Specific antiviral activity demonstrated by TGTP, a member of a new family of interferon-induced GTPases. J Immunol. 1998;161: 2348–2355. 9725230

[43] Früh K, Yang Y. Antigen presentation by MHC class I and its regulation by interferon gamma. Curr Opin Immunol. 1999;11: 76–81. 10047537 10.1016/s0952-7915(99)80014-4

