# Supplementary figures and images for: Modeling for influenza vaccines and adjuvants profile for safety prediction system using gene expression profiling and statistical tools
Source: PLoS One. 2018 Feb 6;13(2):e0191896. doi: 10.1371/journal.pone.0191896 (PMC5800680; doi:10.1371/journal.pone.0191896)

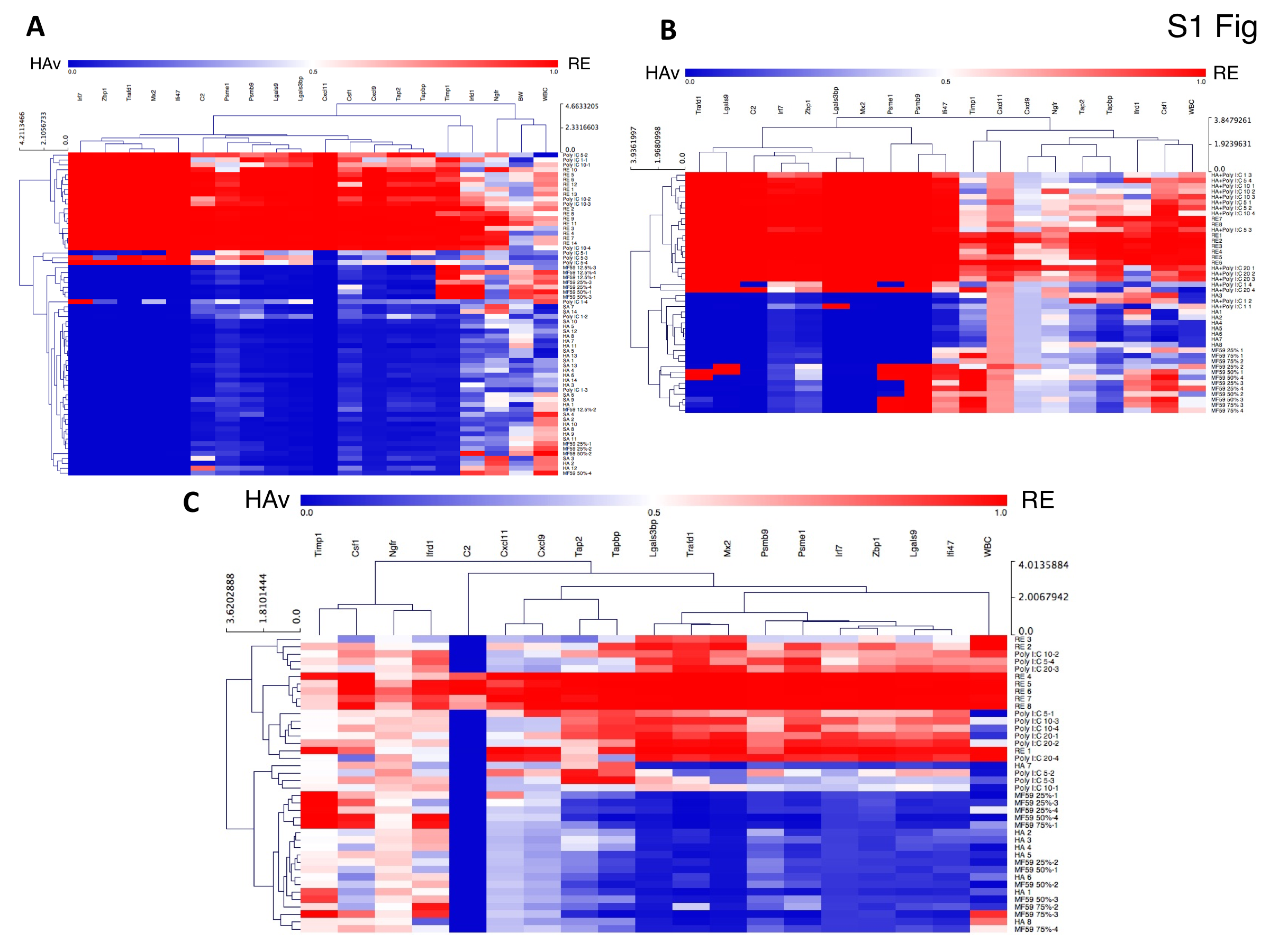

Supplement: S1 Fig — In the heat map, each row is a type of vaccine and each column is a biomarker gene. The heat map is colored by the probability of having RE-like toxicity outcome; blue colors indicate more distinction from RE property, and red colors indicate more closeness to RE property. Vaccines are grouped into clusters of similar predicted profiles. The result shows cases of (a) intranasal, (b) intramuscular, and (c) intraperitoneal vaccination. (TIFF) [file pone.0191896.s001.tiff]
