# Supplementary material for: Modeling for influenza vaccines and adjuvants profile for safety prediction system using gene expression profiling and statistical tools
Source: PLoS One. 2018 Feb 6;13(2):e0191896. doi: 10.1371/journal.pone.0191896 (PMC5800680; doi:10.1371/journal.pone.0191896)
Supplement: S1 Table — (DOCX) [file pone.0191896.s002.docx]

**S1 Table**

Ordinal logistic regression analysis of marker genes in the nasal inoculation group.

|  |  |  | Analysis of Maximum Likelihood Estimation | | | | | | | | | | |
| --- | --- | --- | --- | --- | --- | --- | --- | --- | --- | --- | --- | --- | --- |
| Parameter | Whole-Model Test: Logit *r*^2^ |  | *β_0_* (RE) | | |  | *β_0_* (Poly I:C) | | |  | *β_1_* | | |
|  |  |  | Estimate | S.E. | *p* Value |  | Estimate | S.E. | *p* Value |  | Estimate | S.E. | *p* Value |
|  |  |  |  |  |  |  |  |  |  |  |  |  |  |
| Psme1 | 0.6199 |  | -9.734 | 2.876 | 0.00070 |  | -7.37 | 2.09 | 0.00040 |  | 4.90 | 1.39 | 0.00 |
| Timp1 | 0.693 |  | -6.919 | 2.122 | 0.00110 |  | -4.19 | 1.53 | 0.00630 |  | 0.87 | 0.26 | 0.00 |
| Tap2 | 0.7239 |  | -10.056 | 3.109 | 0.00120 |  | -6.91 | 2.22 | 0.00180 |  | 3.85 | 1.17 | 0.00 |
| C2 | 0.7829 |  | -15.662 | 5.366 | 0.00350 |  | -11.82 | 4.16 | 0.00450 |  | 8.62 | 2.87 | 0.00 |
| Trafd1 | 1 |  | -646.682 | 0.000 | 0.00010 |  | -353.34 | 113739.20 | 0.99750 |  | 260.28 | 0.00 | 0.00 |
| Irf7 | 1 |  | -431.347 | 0.000 | 0.00010 |  | -109.31 | 0.00 | 0.00010 |  | 19.79 | 76511.02 | 1.00 |
| Cxcl11 | 1 |  | -397.445 | 0.000 | 0.00010 |  | -81.92 | 1030313.40 | 0.99990 |  | 2.01 | 6858.49 | 1.00 |
| Psmb9 | 0.8513 |  | -20.370 | 8.880 | 0.02180 |  | -12.00 | 4.50 | 0.00770 |  | 8.48 | 3.49 | 0.02 |
| Cxcl9 | 0.8452 |  | -11.875 | 5.145 | 0.02100 |  | -3.89 | 1.57 | 0.01320 |  | 0.26 | 0.11 | 0.01 |
| Csf1 | 0.6688 |  | -10.071 | 2.930 | 0.00060 |  | -7.64 | 2.30 | 0.00090 |  | 5.14 | 1.49 | 0.00 |
| Ngfr | 0.2589 |  | -4.159 | 1.311 | 0.00150 |  | -3.38 | 1.22 | 0.00570 |  | 2.92 | 0.99 | 0.00 |
| Lgals9 | 0.8958 |  | -19.026 | 90.268 | 0.03510 |  | -9.92 | 4.36 | 0.02300 |  | 5.79 | 2.80 | 0.04 |
| Lgals3bp | 0.8716 |  | -17.959 | 8.037 | 0.02540 |  | -8.22 | 3.52 | 0.01960 |  | 3.53 | 1.54 | 0.02 |
| Zbp1 | 1 |  | -188.750 | 0.000 | 0.00010 |  | -44.93 | 1326812.50 | 1.00000 |  | 12.08 | 250400.92 | 1.00 |
| Mx2 | 1 |  | -332.184 | 0.000 | 0.00010 |  | -82.77 | 0.00 | 0.00010 |  | 24.78 | 0.00 | 0.00 |
| Ifi47 | 1 |  | -259.831 | 0.000 | 0.00010 |  | -119.13 | 0.00 | 0.00010 |  | 78.09 | 0.00 | 0.00 |
| Tapbp | 0.72 |  | -11.339 | 3.505 | 0.00120 |  | -8.02 | 2.46 | 0.00120 |  | 4.85 | 1.44 | 0.00 |
| Irfd1 | 0.3655 |  | -11.203 | 3.596 | 0.00160 |  | -10.21 | 3.46 | 0.00320 |  | 8.94 | 3.04 | 0.00 |
|  |  |  |  |  |  |  |  |  |  |  |  |  |  |
