# Supplementary material for: Modeling for influenza vaccines and adjuvants profile for safety prediction system using gene expression profiling and statistical tools
Source: PLoS One. 2018 Feb 6;13(2):e0191896. doi: 10.1371/journal.pone.0191896 (PMC5800680; doi:10.1371/journal.pone.0191896)
Supplement: S2 Table — (DOCX) [file pone.0191896.s003.docx]

**S2 Table**

Ordinal logistic regression analysis of marker genes in the intramuscular inoculation group.

|  |  |  | Analysis of Maximum Likelihood Estimation | | | | | | | | | | |
| --- | --- | --- | --- | --- | --- | --- | --- | --- | --- | --- | --- | --- | --- |
| Parameter | Whole-Model Test: Logit *r*^2^ |  | *β_0_* (RE) | | |  | *β_1_* (Poly I:C) | | |  | *β_1_* | | |
|  |  |  | Estimate | S.E. | *p* Value |  | Estimate | S.E. | *p* Value |  | Estimate | S.E. | *p* Value |
|  |  |  |  |  |  |  |  |  |  |  |  |  |  |
| Psme1 | 0.6284 |  | -18.883 | 7.114 | 0.00790 |  | -15.996 | 6.248 | 0.01050 |  | 13.418 | 5.269 | 0.01090 |
| Timp1 | 0.0906 |  | -1.318 | 0.736 | 0.07340 |  | -0.389 | 0.686 | 0.57110 |  | 0.640 | 0.460 | 0.16420 |
| Tap2 | 0.3178 |  | -5.472 | 2.135 | 0.01040 |  | -4.076 | 1.931 | 0.03480 |  | 3.373 | 1.565 | 0.03110 |
| C2 | 0.5391 |  | -17.060 | 6.874 | 0.01310 |  | -14.847 | 6.314 | 0.01870 |  | 12.432 | 5.278 | 0.01850 |
| Trafd1 | 0.7102 |  | -19.676 | 7.821 | 0.01190 |  | -16.102 | 6.821 | 0.01820 |  | 12.648 | 5.239 | 0.01580 |
| Irf7 | 0.5571 |  | -5.086 | 1.825 | 0.00530 |  | -2.540 | 1.146 | 0.02670 |  | 0.446 | 0.171 | 0.00920 |
| Cxcl11 | 0.3075 |  | -2.122 | 0.833 | 0.01090 |  | -0.665 | 0.610 | 0.27590 |  | 0.138 | 0.062 | 0.02740 |
| Psmb9 | 0.4763 |  | -7.868 | 2.921 | 0.00710 |  | -5.897 | 2.511 | 0.01890 |  | 4.265 | 1.809 | 0.01840 |
| Cxcl9 | 0.3918 |  | -2.754 | 1.033 | 0.00770 |  | -1.021 | 0.659 | 0.12150 |  | 0.241 | 0.099 | 0.01530 |
| Csf1 | 0.2681 |  | -7.315 | 3.190 | 0.02190 |  | -6.030 | 3.034 | 0.04690 |  | 5.853 | 2.846 | 0.03970 |
| Ngfr | 0.0176 |  | -1.235 | 1.051 | 0.23990 |  | -0.400 | 1.018 | 0.69450 |  | 0.735 | 0.848 | 0.38620 |
| Lgals9 | 0.5683 |  | -8.502 | 3.019 | 0.00490 |  | -6.026 | 2.332 | 0.00980 |  | 3.887 | 1.480 | 0.00870 |
| Lgals3bp | 0.7146 |  | -10.264 | 3.924 | 0.00890 |  | -6.224 | 2.633 | 0.01810 |  | 9.090 | 1.216 | 0.01100 |
| Zbp1 | 0.6281 |  | -5.966 | 2.102 | 0.00450 |  | -3.110 | 1.387 | 0.02500 |  | 0.802 | 0.313 | 0.01030 |
| Mx2 | 0.754 |  | -9.258 | 3.815 | 0.01520 |  | -5.021 | 2.729 | 0.06540 |  | 1.247 | 0.547 | 0.02250 |
| Ifi47 | 0.5682 |  | -7.455 | 2.685 | 0.00550 |  | -5.016 | 2.021 | 0.01310 |  | 2.820 | 1.095 | 0.01000 |
| Tapbp | 0.4659 |  | -11.546 | 4.441 | 0.00930 |  | -9.582 | 3.970 | 0.01580 |  | 8.466 | 3.494 | 0.01540 |
| Irfd1 | 0.1513 |  | -6.301 | 2.853 | 0.02720 |  | -5.270 | 2.749 | 0.05520 |  | 5.195 | 2.537 | 0.04060 |
|  |  |  |  |  |  |  |  |  |  |  |  |  |  |
