# Supplementary material for: Modeling for influenza vaccines and adjuvants profile for safety prediction system using gene expression profiling and statistical tools
Source: PLoS One. 2018 Feb 6;13(2):e0191896. doi: 10.1371/journal.pone.0191896 (PMC5800680; doi:10.1371/journal.pone.0191896)
Supplement: S3 Table — (DOCX) [file pone.0191896.s004.docx]

**S3 Table**

Ordinal logistic regression analysis of marker genes in the intraperitoneal inoculation group.

|  |  |  | Analysis of Maximum Likelihood Estimation | | | | | | | | | | |
| --- | --- | --- | --- | --- | --- | --- | --- | --- | --- | --- | --- | --- | --- |
| Parameter | Whole-Model Test: Logit *r*^2^ |  | *β_0_* (RE) | | |  | *β_0_* (Poly I:C) | | |  | *β_0_* | | |
|  |  |  | Estimate | S.E. | *p* Value |  | Estimate | S.E. | *p* Value |  | Estimate | S.E. | *p* Value |
|  |  |  |  |  |  |  |  |  |  |  |  |  |  |
| Psme1 | 1 |  | -577.762 | 0.000 | 0.00010 |  | -361.079 | 94735.809 | 0.99700 |  | 298.202 | 0.000 | 0.00010 |
| Timp1 | 0.2976 |  | -5.479 | 1.940 | 0.00480 |  | -4.043 | 1.719 | 0.01870 |  | 2.344 | 0.901 | 0.00930 |
| Tap2 | 0.3418 |  | -4.687 | 1.584 | 0.00310 |  | -3.155 | 1.312 | 0.01620 |  | 2.094 | 0.751 | 0.00530 |
| C2 | 0.9264 |  | -191.864 | 275.498 | 0.48620 |  | -109.467 | 158.472 | 0.48970 |  | 52.625 | 75.666 | 0.48680 |
| Trafd1 | 1 |  | -357.143 | 0.000 | 0.00010 |  | -210.652 | 195017.160 | 0.99910 |  | 149.106 | 0.000 | 0.00010 |
| Irf7 | 0.7286 |  | -13.868 | 8.629 | 0.10800 |  | -3.193 | 1.493 | 0.03240 |  | 0.473 | 0.298 | 0.11290 |
| Cxcl11 | 0.3066 |  | -2.253 | 0.868 | 0.00940 |  | -0.835 | 0.635 | 0.18910 |  | 0.038 | 0.016 | 0.01970 |
| Psmb9 | 1 |  | -3513.004 | 223045.900 | 0.98740 |  | -1631.654 | 15599.343 | 0.91670 |  | 1318.162 | 0.000 | 0.00010 |
| Cxcl9 | 0.1885 |  | -1.677 | 0.717 | 0.01930 |  | -0.531 | 0.601 | 0.37750 |  | 0.043 | 0.022 | 0.04640 |
| Csf1 | 0.6125 |  | -16.924 | 6.209 | 0.00640 |  | -13.967 | 5.351 | 0.00900 |  | 10.618 | 3.931 | 0.00690 |
| Ngfr | 0.0806 |  | -2.183 | 1.155 | 0.05880 |  | -1.236 | 1.082 | 0.25330 |  | 0.827 | 0.525 | 0.11520 |
| Lgals9 | 1 |  | -269.412 | 0.000 | 0.00010 |  | -145.867 | 309646.170 | 0.99960 |  | 99.801 | 0.000 | 0.00010 |
| Lgals3bp | 1 |  | -225.806 | 941512.660 | 0.99980 |  | -78.357 | 771537.590 | 0.99990 |  | 42.739 | 0.000 | 0.01000 |
| Zbp1 | 0.8329 |  | -17.775 | 10.092 | 0.07820 |  | -4.345 | 2.206 | 0.04890 |  | 0.949 | 0.521 | 0.06870 |
| Mx2 | 1 |  | -419.187 | 859908.410 | 0.99960 |  | -109.348 | 0.000 | 0.00010 |  | 28.459 | 0.000 | 0.00010 |
| Ifi47 | 0.8285 |  | -18.696 | 11.289 | 0.09770 |  | -7.081 | 3.535 | 0.04520 |  | 4.197 | 2.517 | 0.09540 |
| Tapbp | 0.5494 |  | -11.296 | 3.788 | 0.00290 |  | -8.518 | 3.062 | 0.00540 |  | 5.862 | 1.952 | 0.02700 |
| Irfd1 | 0.4351 |  | -16.071 | 5.594 | 0.00410 |  | -14.179 | 5.197 | 0.00640 |  | 12.460 | 4.494 | 0.00560 |
|  |  |  |  |  |  |  |  |  |  |  |  |  |  |
