# Supplementary material for: Modeling for influenza vaccines and adjuvants profile for safety prediction system using gene expression profiling and statistical tools
Source: PLoS One. 2018 Feb 6;13(2):e0191896. doi: 10.1371/journal.pone.0191896 (PMC5800680; doi:10.1371/journal.pone.0191896)
Supplement: S4 Table — Data are presented as the mean ± S.D. (DOCX) [file pone.0191896.s005.docx]

**S4 Table**

The marker genes expression profiles in Advax group

Data are presented as the mean ± S.D.

| Route | Vaccine and adjuvant | Marker genes | | | | | | | | | | | | | | | | | |
| --- | --- | --- | --- | --- | --- | --- | --- | --- | --- | --- | --- | --- | --- | --- | --- | --- | --- | --- | --- |
|  |  | *Psme1* | | | *Timp1* | | | *Tap2* | | | *C2* | | | *Trafd1* | | | *Irf7* | | |
| ip | SA | 0.13518 | ± | 0.00953 | 0.00860 | ± | 0.00109 | 0.00607 | ± | 0.00125 | 0.02141 | ± | 0.00179 | 0.05591 | ± | 0.00287 | 0.00727 | ± | 0.00159 |
|  | HAv | 0.12849 | ± | 0.01361 | 0.00619 | ± | 0.00095 | 0.00641 | ± | 0.00352 | 0.01567 | ± | 0.00134 | 0.05270 | ± | 0.00462 | 0.00741 | ± | 0.00196 |
|  | Advax 25 | 0.15331 | ± | 0.01443 | 0.02034 | ± | 0.01121 | 0.00772 | ± | 0.00110 | 0.02316 | ± | 0.00612 | 0.06764 | ± | 0.00442 | 0.01484 | ± | 0.00517 |
|  | Advax 50 | 0.15975 | ± | 0.00567 | 0.03116 | ± | 0.01359 | 0.00869 | ± | 0.00141 | 0.02691 | ± | 0.00238 | 0.07525 | ± | 0.00667 | 0.01563 | ± | 0.00568 |
|  | Advax 75 | 0.14778 | ± | 0.01531 | 0.01923 | ± | 0.00471 | 0.00700 | ± | 0.00018 | 0.02331 | ± | 0.00088 | 0.06548 | ± | 0.00666 | 0.01273 | ± | 0.00182 |
|  | RE | 0.30588 | ± | 0.03751 | 0.01486 | ± | 0.00548 | 0.01258 | ± | 0.00424 | 0.07015 | ± | 0.01506 | 0.16971 | ± | 0.01789 | 0.24998 | ± | 0.05598 |
|  |  |  |  |  |  |  |  |  |  |  |  |  |  |  |  |  |  |  |  |
| im | SA | 0.13550 | ± | 0.00699 | 0.00709 | ± | 0.00111 | 0.00497 | ± | 0.00181 | 0.02268 | ± | 0.00369 | 0.05492 | ± | 0.00485 | 0.00785 | ± | 0.00192 |
|  | HAv | 0.13855 | ± | 0.00534 | 0.00787 | ± | 0.00096 | 0.00579 | ± | 0.00073 | 0.02393 | ± | 0.00270 | 0.05292 | ± | 0.00402 | 0.00662 | ± | 0.00066 |
|  | Advax 25 | 0.14546 | ± | 0.00379 | 0.00779 | ± | 0.00088 | 0.00521 | ± | 0.00164 | 0.02510 | ± | 0.00116 | 0.05832 | ± | 0.00196 | 0.00669 | ± | 0.00079 |
|  | Advax 50 | 0.15210 | ± | 0.00982 | 0.00866 | ± | 0.00036 | 0.00624 | ± | 0.00086 | 0.02353 | ± | 0.00220 | 0.05787 | ± | 0.00536 | 0.00785 | ± | 0.00190 |
|  | Advax 75 | 0.13312 | ± | 0.00792 | 0.00594 | ± | 0.00129 | 0.00678 | ± | 0.00352 | 0.02225 | ± | 0.00284 | 0.05936 | ± | 0.00712 | 0.00955 | ± | 0.00436 |
|  | RE | 0.32303 | ± | 0.02124 | 0.00936 | ± | 0.00244 | 0.01627 | ± | 0.00589 | 0.05116 | ± | 0.00860 | 0.18144 | ± | 0.01597 | 0.27883 | ± | 0.05155 |
|  |  |  |  |  |  |  |  |  |  |  |  |  |  |  |  |  |  |  |  |
| in | SA | 0.14188 | ± | 0.00486 | 0.01184 | ± | 0.00618 | 0.00459 | ± | 0.00062 | 0.02512 | ± | 0.00363 | 0.05355 | ± | 0.00306 | 0.00664 | ± | 0.00069 |
|  | HAv | 0.14567 | ± | 0.00768 | 0.00956 | ± | 0.00173 | 0.00570 | ± | 0.00144 | 0.02332 | ± | 0.00197 | 0.05837 | ± | 0.00541 | 0.00699 | ± | 0.00146 |
|  | Advax 12.5 | 0.14613 | ± | 0.00459 | 0.10865 | ± | 0.06695 | 0.00426 | ± | 0.00099 | 0.02244 | ± | 0.00241 | 0.05399 | ± | 0.00605 | 0.00778 | ± | 0.00135 |
|  | Advax 25 | 0.13708 | ± | 0.03758 | 0.17476 | ± | 0.30197 | 0.00413 | ± | 0.00125 | 0.02068 | ± | 0.00916 | 0.05153 | ± | 0.01820 | 0.00607 | ± | 0.00088 |
|  | Advax 50 | 0.13863 | ± | 0.02330 | 0.15774 | ± | 0.17340 | 0.00457 | ± | 0.00139 | 0.02232 | ± | 0.00633 | 0.05430 | ± | 0.01578 | 0.00600 | ± | 0.00054 |
|  | RE | 0.24039 | ± | 0.08524 | 0.05088 | ± | 0.04366 | 0.00905 | ± | 0.00544 | 0.03758 | ± | 0.01736 | 0.12435 | ± | 0.05351 | 0.11658 | ± | 0.10682 |
