# Supplementary material for: Modeling for influenza vaccines and adjuvants profile for safety prediction system using gene expression profiling and statistical tools
Source: PLoS One. 2018 Feb 6;13(2):e0191896. doi: 10.1371/journal.pone.0191896 (PMC5800680; doi:10.1371/journal.pone.0191896)
Supplement: S5 Table — Data are presented as the mean ± S.D. (DOCX) [file pone.0191896.s006.docx]

**S5 Table**

The marker genes expression profiles in Advax group

Data are presented as the mean ± S.D.

| Route | Vaccine and adjuvant | Marker genes | | | | | | | | | | | | | | | | | |
| --- | --- | --- | --- | --- | --- | --- | --- | --- | --- | --- | --- | --- | --- | --- | --- | --- | --- | --- | --- |
|  |  | *Cxcl11* | | | *Psmb9* | | | *Cxcl9* | | | *Csf1* | | | *Ngfr* | | | *Lgals9* | | |
| ip | SA | 0.00012 | ± | 0.00005 | 0.07396 | ± | 0.00895 | 0.00021 | ± | 0.00004 | 0.00596 | ± | 0.00065 | 0.00026 | ± | 0.00006 | 0.05075 | ± | 0.00277 |
|  | HAv | 0.00014 | ± | 0.00003 | 0.06350 | ± | 0.00412 | 0.00016 | ± | 0.00006 | 0.00566 | ± | 0.00108 | 0.00048 | ± | 0.00020 | 0.04618 | ± | 0.00541 |
|  | Advax 25 | 0.00122 | ± | 0.00184 | 0.09404 | ± | 0.01515 | 0.00191 | ± | 0.00252 | 0.00820 | ± | 0.00075 | 0.00053 | ± | 0.00012 | 0.06049 | ± | 0.00732 |
|  | Advax 50 | 0.00043 | ± | 0.00045 | 0.09890 | ± | 0.01221 | 0.00088 | ± | 0.00095 | 0.00886 | ± | 0.00099 | 0.00069 | ± | 0.00016 | 0.06141 | ± | 0.01021 |
|  | Advax 75 | 0.00020 | ± | 0.00006 | 0.08410 | ± | 0.00957 | 0.00028 | ± | 0.00013 | 0.00774 | ± | 0.00099 | 0.00046 | ± | 0.00014 | 0.05632 | ± | 0.00411 |
|  | RE | 0.00728 | ± | 0.01044 | 0.21975 | ± | 0.01807 | 0.00970 | ± | 0.01342 | 0.00956 | ± | 0.00042 | 0.00080 | ± | 0.00014 | 0.18067 | ± | 0.01672 |
|  |  |  |  |  |  |  |  |  |  |  |  |  |  |  |  |  |  |  |  |
| im | SA | 0.00014 | ± | 0.00003 | 0.06890 | ± | 0.00480 | 0.00028 | ± | 0.00009 | 0.00609 | ± | 0.00081 | 0.00049 | ± | 0.00020 | 0.05230 | ± | 0.00308 |
|  | HAv | 0.00011 | ± | 0.00003 | 0.06497 | ± | 0.00288 | 0.00029 | ± | 0.00005 | 0.00676 | ± | 0.00034 | 0.00067 | ± | 0.00018 | 0.04724 | ± | 0.00280 |
|  | Advax 25 | 0.00012 | ± | 0.00002 | 0.07522 | ± | 0.00791 | 0.00025 | ± | 0.00007 | 0.00677 | ± | 0.00110 | 0.00061 | ± | 0.00018 | 0.05025 | ± | 0.00218 |
|  | Advax 50 | 0.00014 | ± | 0.00002 | 0.08630 | ± | 0.01146 | 0.00021 | ± | 0.00007 | 0.00790 | ± | 0.00172 | 0.00048 | ± | 0.00011 | 0.05298 | ± | 0.00165 |
|  | Advax 75 | 0.00016 | ± | 0.00007 | 0.09106 | ± | 0.01996 | 0.00023 | ± | 0.00012 | 0.00674 | ± | 0.00195 | 0.00068 | ± | 0.00035 | 0.04983 | ± | 0.00875 |
|  | RE | 0.00159 | ± | 0.00052 | 0.23426 | ± | 0.01066 | 0.00269 | ± | 0.00078 | 0.01131 | ± | 0.00099 | 0.00080 | ± | 0.00028 | 0.19494 | ± | 0.01921 |
|  |  |  |  |  |  |  |  |  |  |  |  |  |  |  |  |  |  |  |  |
| in | SA | 0.00015 | ± | 0.00003 | 0.07162 | ± | 0.00671 | 0.00031 | ± | 0.00014 | 0.00737 | ± | 0.00075 | 0.00079 | ± | 0.00024 | 0.04536 | ± | 0.00134 |
|  | HAv | 0.00018 | ± | 0.00004 | 0.07212 | ± | 0.00195 | 0.00018 | ± | 0.00007 | 0.00722 | ± | 0.00094 | 0.00078 | ± | 0.00020 | 0.04770 | ± | 0.00264 |
|  | Advax 12.5 | 0.00077 | ± | 0.00080 | 0.06669 | ± | 0.00985 | 0.00043 | ± | 0.00015 | 0.00797 | ± | 0.00078 | 0.00068 | ± | 0.00017 | 0.04485 | ± | 0.00397 |
|  | Advax 25 | 0.00023 | ± | 0.00010 | 0.06410 | ± | 0.03098 | 0.00034 | ± | 0.00012 | 0.00768 | ± | 0.00228 | 0.00056 | ± | 0.00008 | 0.04171 | ± | 0.01002 |
|  | Advax 50 | 0.00019 | ± | 0.00007 | 0.05726 | ± | 0.02146 | 0.00031 | ± | 0.00016 | 0.00835 | ± | 0.00175 | 0.00060 | ± | 0.00019 | 0.04538 | ± | 0.00900 |
|  | RE | 0.01513 | ± | 0.02224 | 0.13402 | ± | 0.05307 | 0.00689 | ± | 0.01072 | 0.00919 | ± | 0.00348 | 0.00045 | ± | 0.00011 | 0.12481 | ± | 0.06679 |
