# Supplementary material for: Modeling for influenza vaccines and adjuvants profile for safety prediction system using gene expression profiling and statistical tools
Source: PLoS One. 2018 Feb 6;13(2):e0191896. doi: 10.1371/journal.pone.0191896 (PMC5800680; doi:10.1371/journal.pone.0191896)
Supplement: S6 Table — Data are presented as the mean ± S.D. (DOCX) [file pone.0191896.s007.docx]

**S6 Table**

The marker genes expression profiles in Advax group

Data are presented as the mean ± S.D.

| Route | Vaccine and adjuvant | Marker genes | | | | | | | | | | | | | | | | | |
| --- | --- | --- | --- | --- | --- | --- | --- | --- | --- | --- | --- | --- | --- | --- | --- | --- | --- | --- | --- |
|  |  | *Lgals3bp* | | | *Zbp1* | | | *Mx2* | | | *Ifi47* | | | *Tapbp* | | | *Ifrd1* | | |
| ip | SA | 0.06019 | ± | 0.00785 | 0.00833 | ± | 0.00197 | 0.00339 | ± | 0.00051 | 0.03155 | ± | 0.00371 | 0.06629 | ± | 0.00757 | 0.11130 | ± | 0.00518 |
|  | HAv | 0.05847 | ± | 0.00701 | 0.00799 | ± | 0.00205 | 0.00337 | ± | 0.00037 | 0.02968 | ± | 0.00397 | 0.06089 | ± | 0.01745 | 0.10694 | ± | 0.00745 |
|  | Advax 25 | 0.07439 | ± | 0.00888 | 0.02580 | ± | 0.00901 | 0.00775 | ± | 0.00291 | 0.06032 | ± | 0.01018 | 0.07261 | ± | 0.00438 | 0.12612 | ± | 0.00745 |
|  | Advax 50 | 0.07729 | ± | 0.01241 | 0.02669 | ± | 0.00799 | 0.00750 | ± | 0.00352 | 0.06278 | ± | 0.00743 | 0.07555 | ± | 0.00731 | 0.12879 | ± | 0.00343 |
|  | Advax 75 | 0.06697 | ± | 0.00340 | 0.01874 | ± | 0.00322 | 0.00582 | ± | 0.00090 | 0.05307 | ± | 0.00554 | 0.06684 | ± | 0.00179 | 0.11569 | ± | 0.01355 |
|  | RE | 0.41994 | ± | 0.06391 | 0.17876 | ± | 0.03204 | 0.06494 | ± | 0.00890 | 0.16203 | ± | 0.02523 | 0.12430 | ± | 0.01319 | 0.16336 | ± | 0.01935 |
|  |  |  |  |  |  |  |  |  |  |  |  |  |  |  |  |  |  |  |  |
| im | SA | 0.05764 | ± | 0.00639 | 0.00922 | ± | 0.00128 | 0.00413 | ± | 0.00116 | 0.03344 | ± | 0.00342 | 0.05517 | ± | 0.01453 | 0.10995 | ± | 0.00939 |
|  | HAv | 0.05943 | ± | 0.00067 | 0.00880 | ± | 0.00113 | 0.00362 | ± | 0.00123 | 0.02970 | ± | 0.00094 | 0.05885 | ± | 0.00355 | 0.09934 | ± | 0.00543 |
|  | Advax 25 | 0.06287 | ± | 0.00500 | 0.01063 | ± | 0.00063 | 0.00385 | ± | 0.00032 | 0.03680 | ± | 0.00289 | 0.06110 | ± | 0.00819 | 0.11504 | ± | 0.00715 |
|  | Advax 50 | 0.06450 | ± | 0.00857 | 0.01243 | ± | 0.00219 | 0.00411 | ± | 0.00053 | 0.04228 | ± | 0.00535 | 0.06470 | ± | 0.00826 | 0.12758 | ± | 0.00983 |
|  | Advax 75 | 0.06633 | ± | 0.01601 | 0.01550 | ± | 0.00554 | 0.00406 | ± | 0.00179 | 0.04720 | ± | 0.01303 | 0.06260 | ± | 0.01705 | 0.12054 | ± | 0.00957 |
|  | RE | 0.43273 | ± | 0.03265 | 0.20159 | ± | 0.01930 | 0.06852 | ± | 0.00737 | 0.18576 | ± | 0.01526 | 0.14966 | ± | 0.03158 | 0.14634 | ± | 0.01077 |
|  |  |  |  |  |  |  |  |  |  |  |  |  |  |  |  |  |  |  |  |
| in | SA | 0.06377 | ± | 0.00329 | 0.01057 | ± | 0.00101 | 0.00358 | ± | 0.00038 | 0.02943 | ± | 0.00353 | 0.05648 | ± | 0.00253 | 0.10575 | ± | 0.00685 |
|  | HAv | 0.06047 | ± | 0.00725 | 0.00904 | ± | 0.00171 | 0.00335 | ± | 0.00024 | 0.02869 | ± | 0.00239 | 0.06271 | ± | 0.01416 | 0.10548 | ± | 0.01725 |
|  | Advax 12.5 | 0.06165 | ± | 0.00460 | 0.01290 | ± | 0.00224 | 0.00400 | ± | 0.00047 | 0.02906 | ± | 0.00173 | 0.05523 | ± | 0.00420 | 0.11406 | ± | 0.00911 |
|  | Advax 25 | 0.05713 | ± | 0.00667 | 0.01074 | ± | 0.00108 | 0.00340 | ± | 0.00073 | 0.02767 | ± | 0.00898 | 0.04877 | ± | 0.01364 | 0.14840 | ± | 0.04885 |
|  | Advax 50 | 0.05406 | ± | 0.00518 | 0.00915 | ± | 0.00156 | 0.00346 | ± | 0.00138 | 0.02728 | ± | 0.01021 | 0.05053 | ± | 0.01125 | 0.17074 | ± | 0.03641 |
|  | RE | 0.25450 | ± | 0.11836 | 0.11832 | ± | 0.10890 | 0.05146 | ± | 0.04305 | 0.10643 | ± | 0.07159 | 0.10271 | ± | 0.04165 | 0.11850 | ± | 0.01079 |
