# Supplementary material for: Modeling for influenza vaccines and adjuvants profile for safety prediction system using gene expression profiling and statistical tools
Source: PLoS One. 2018 Feb 6;13(2):e0191896. doi: 10.1371/journal.pone.0191896 (PMC5800680; doi:10.1371/journal.pone.0191896)
Supplement: S7 Table — Data are presented as the mean ± S.D. (DOCX) [file pone.0191896.s008.docx]

**S7 Table**

The marker genes expression profiles in Poly I:C group

Data are presented as the mean ± S.D.

| Route | Vaccine and adjuvant | Marker genes | | | | | | | | | | | | | | | | | |
| --- | --- | --- | --- | --- | --- | --- | --- | --- | --- | --- | --- | --- | --- | --- | --- | --- | --- | --- | --- |
|  |  | *Psme1* | | | *Timp1* | | | *Tap2* | | | *C2* | | | *Trafd1* | | | *Irf7* | | |
| ip | SA | 0.15638 | ± | 0.00983 | 0.00735 | ± | 0.00097 | 0.00613 | ± | 0.00153 | 0.01733 | ± | 0.00109 | 0.06232 | ± | 0.00561 | 0.00708 | ± | 0.00129 |
|  | HAv | 0.16227 | ± | 0.00752 | 0.01024 | ± | 0.00164 | 0.00684 | ± | 0.00219 | 0.02537 | ± | 0.00634 | 0.07079 | ± | 0.00482 | 0.00695 | ± | 0.00157 |
|  | Poly I:C-1 | 0.17102 | ± | 0.03443 | 0.00916 | ± | 0.00233 | 0.01016 | ± | 0.00700 | 0.02563 | ± | 0.00931 | 0.08643 | ± | 0.01524 | 0.04059 | ± | 0.02492 |
|  | Poly I:C-5 | 0.24493 | ± | 0.00911 | 0.01216 | ± | 0.00140 | 0.00981 | ± | 0.00288 | 0.04611 | ± | 0.00520 | 0.12822 | ± | 0.00671 | 0.11424 | ± | 0.02503 |
|  | Poly I:C-10 | 0.24596 | ± | 0.01360 | 0.01159 | ± | 0.00253 | 0.00969 | ± | 0.00133 | 0.04623 | ± | 0.00549 | 0.12379 | ± | 0.00975 | 0.14241 | ± | 0.03519 |
|  | Poly I:C-20 | 0.24480 | ± | 0.03563 | 0.01903 | ± | 0.00453 | 0.01179 | ± | 0.00482 | 0.05425 | ± | 0.01154 | 0.12317 | ± | 0.01714 | 0.16621 | ± | 0.08037 |
|  | RE | 0.40363 | ± | 0.03539 | 0.02045 | ± | 0.00657 | 0.02241 | ± | 0.00222 | 0.09221 | ± | 0.01695 | 0.24212 | ± | 0.02459 | 0.40356 | ± | 0.02345 |
|  |  |  |  |  |  |  |  |  |  |  |  |  |  |  |  |  |  |  |  |
| im | SA | 0.14739 | ± | 0.00160 | 0.00856 | ± | 0.00149 | 0.00788 | ± | 0.00277 | 0.02326 | ± | 0.00172 | 0.05844 | ± | 0.00330 | 0.00920 | ± | 0.00110 |
|  | HAv | 0.14387 | ± | 0.01079 | 0.01647 | ± | 0.01781 | 0.00596 | ± | 0.00108 | 0.02226 | ± | 0.00389 | 0.05907 | ± | 0.00542 | 0.00695 | ± | 0.00216 |
|  | Poly I:C-1 | 0.18356 | ± | 0.01995 | 0.00945 | ± | 0.00260 | 0.01054 | ± | 0.00339 | 0.02709 | ± | 0.00564 | 0.07761 | ± | 0.00818 | 0.03736 | ± | 0.00802 |
|  | Poly I:C-5 | 0.18652 | ± | 0.01448 | 0.00871 | ± | 0.00059 | 0.00852 | ± | 0.00132 | 0.03013 | ± | 0.00109 | 0.08284 | ± | 0.00459 | 0.04936 | ± | 0.01421 |
|  | Poly I:C-10 | 0.19691 | ± | 0.00873 | 0.01189 | ± | 0.00730 | 0.00818 | ± | 0.00137 | 0.03042 | ± | 0.00081 | 0.08852 | ± | 0.00408 | 0.07157 | ± | 0.01254 |
|  | Poly I:C-20 | 0.23107 | ± | 0.00311 | 0.01216 | ± | 0.00259 | 0.01134 | ± | 0.00294 | 0.04156 | ± | 0.00184 | 0.11200 | ± | 0.00918 | 0.13190 | ± | 0.02867 |
|  | RE | 0.39527 | ± | 0.05156 | 0.01609 | ± | 0.00239 | 0.01932 | ± | 0.00760 | 0.07709 | ± | 0.02456 | 0.20540 | ± | 0.03254 | 0.33930 | ± | 0.10927 |
|  |  |  |  |  |  |  |  |  |  |  |  |  |  |  |  |  |  |  |  |
| in | SA | 0.16146 | ± | 0.00732 | 0.01371 | ± | 0.00653 | 0.00619 | ± | 0.00162 | 0.02737 | ± | 0.00710 | 0.06435 | ± | 0.00325 | 0.01057 | ± | 0.00071 |
|  | HAv | 0.16448 | ± | 0.01283 | 0.01320 | ± | 0.00560 | 0.00581 | ± | 0.00139 | 0.02535 | ± | 0.00171 | 0.06334 | ± | 0.00410 | 0.00824 | ± | 0.00107 |
|  | Poly I:C-1 | 0.21523 | ± | 0.03884 | 0.05507 | ± | 0.05211 | 0.00958 | ± | 0.00267 | 0.03129 | ± | 0.00509 | 0.08276 | ± | 0.01561 | 0.05257 | ± | 0.03500 |
|  | Poly I:C-5 | 0.26874 | ± | 0.03192 | 0.04891 | ± | 0.01561 | 0.01148 | ± | 0.00472 | 0.03814 | ± | 0.00569 | 0.09364 | ± | 0.00608 | 0.07501 | ± | 0.02171 |
|  | Poly I:C-10 | 0.32858 | ± | 0.07010 | 0.11688 | ± | 0.01835 | 0.01604 | ± | 0.00319 | 0.04410 | ± | 0.00800 | 0.12423 | ± | 0.02471 | 0.17631 | ± | 0.05629 |
|  | Poly I:C-20 | 0.25863 | ± | 0.07745 | 0.07690 | ± | 0.05864 | 0.01636 | ± | 0.00687 | 0.04164 | ± | 0.01245 | 0.11371 | ± | 0.03631 | 0.15048 | ± | 0.09954 |
|  | RE | 0.48704 | ± | 0.08156 | 0.23975 | ± | 0.06247 | 0.04492 | ± | 0.01512 | 0.11556 | ± | 0.04390 | 0.33104 | ± | 0.08190 | 0.48649 | ± | 0.09389 |
