# Supplementary material for: Modeling for influenza vaccines and adjuvants profile for safety prediction system using gene expression profiling and statistical tools
Source: PLoS One. 2018 Feb 6;13(2):e0191896. doi: 10.1371/journal.pone.0191896 (PMC5800680; doi:10.1371/journal.pone.0191896)
Supplement: S8 Table — Data are presented as the mean ± S.D. (DOCX) [file pone.0191896.s009.docx]

**S8 Table**

The marker genes expression profiles in Poly I:C group

Data are presented as the mean ± S.D.

| Route | Vaccine and adjuvant | Marker genes | | | | | | | | | | | | | | | | | |
| --- | --- | --- | --- | --- | --- | --- | --- | --- | --- | --- | --- | --- | --- | --- | --- | --- | --- | --- | --- |
|  |  | *Cxcl11* | | | *Psmb9* | | | *Cxcl9* | | | *Csf1* | | | *Ngfr* | | | *Lgals9* | | |
| ip | SA | 0.00010 | ± | 0.00007 | 0.08897 | ± | 0.00707 | 0.00045 | ± | 0.00064 | 0.00556 | ± | 0.00080 | 0.00031 | ± | 0.00007 | 0.05581 | ± | 0.00339 |
|  | HAv | 0.00006 | ± | 0.00008 | 0.09042 | ± | 0.01350 | 0.00014 | ± | 0.00008 | 0.00636 | ± | 0.00154 | 0.00051 | ± | 0.00019 | 0.06128 | ± | 0.00457 |
|  | Poly I:C-1 | 0.00008 | ± | 0.00006 | 0.10356 | ± | 0.02257 | 0.00030 | ± | 0.00018 | 0.00725 | ± | 0.00032 | 0.00028 | ± | 0.00009 | 0.08429 | ± | 0.03282 |
|  | Poly I:C-5 | 0.00095 | ± | 0.00113 | 0.16068 | ± | 0.00650 | 0.00151 | ± | 0.00126 | 0.00895 | ± | 0.00079 | 0.00062 | ± | 0.00025 | 0.13481 | ± | 0.00695 |
|  | Poly I:C-10 | 0.00066 | ± | 0.00025 | 0.14950 | ± | 0.01210 | 0.00292 | ± | 0.00108 | 0.00836 | ± | 0.00084 | 0.00070 | ± | 0.00032 | 0.12918 | ± | 0.00968 |
|  | Poly I:C-20 | 0.00495 | ± | 0.00343 | 0.17210 | ± | 0.05084 | 0.01849 | ± | 0.01587 | 0.00790 | ± | 0.00104 | 0.00089 | ± | 0.00043 | 0.12019 | ± | 0.01821 |
|  | RE | 0.01889 | ± | 0.01185 | 0.32262 | ± | 0.01842 | 0.02106 | ± | 0.01438 | 0.01265 | ± | 0.00062 | 0.00088 | ± | 0.00020 | 0.27176 | ± | 0.01659 |
|  |  |  |  |  |  |  |  |  |  |  |  |  |  |  |  |  |  |  |  |
| im | SA | 0.00011 | ± | 0.00004 | 0.08211 | ± | 0.00509 | 0.00021 | ± | 0.00012 | 0.00621 | ± | 0.00134 | 0.00083 | ± | 0.00013 | 0.05349 | ± | 0.00185 |
|  | HAv | 0.00013 | ± | 0.00004 | 0.07201 | ± | 0.01676 | 0.00020 | ± | 0.00004 | 0.00664 | ± | 0.00048 | 0.00078 | ± | 0.00021 | 0.04989 | ± | 0.00675 |
|  | Poly I:C-1 | 0.00090 | ± | 0.00095 | 0.11351 | ± | 0.01981 | 0.00121 | ± | 0.00107 | 0.00754 | ± | 0.00065 | 0.00090 | ± | 0.00042 | 0.08124 | ± | 0.00802 |
|  | Poly I:C-5 | 0.00016 | ± | 0.00001 | 0.10518 | ± | 0.00712 | 0.00048 | ± | 0.00013 | 0.00768 | ± | 0.00063 | 0.00094 | ± | 0.00013 | 0.08608 | ± | 0.00738 |
|  | Poly I:C-10 | 0.00143 | ± | 0.00209 | 0.12826 | ± | 0.02042 | 0.00126 | ± | 0.00124 | 0.00693 | ± | 0.00103 | 0.00086 | ± | 0.00028 | 0.10163 | ± | 0.00979 |
|  | Poly I:C-20 | 0.00209 | ± | 0.00211 | 0.15679 | ± | 0.00830 | 0.00213 | ± | 0.00144 | 0.00733 | ± | 0.00037 | 0.00117 | ± | 0.00053 | 0.13036 | ± | 0.00434 |
|  | RE | 0.00426 | ± | 0.00197 | 0.30430 | ± | 0.02373 | 0.00698 | ± | 0.00429 | 0.01300 | ± | 0.00262 | 0.00144 | ± | 0.00049 | 0.26249 | ± | 0.04483 |
|  |  |  |  |  |  |  |  |  |  |  |  |  |  |  |  |  |  |  |  |
| in | SA | 0.00016 | ± | 0.00015 | 0.08990 | ± | 0.01467 | 0.00043 | ± | 0.00035 | 0.00715 | ± | 0.00096 | 0.00097 | ± | 0.00063 | 0.05353 | ± | 0.00266 |
|  | HAv | 0.00012 | ± | 0.00003 | 0.08600 | ± | 0.01322 | 0.00027 | ± | 0.00017 | 0.00694 | ± | 0.00083 | 0.00119 | ± | 0.00044 | 0.05214 | ± | 0.00475 |
|  | Poly I:C-1 | 0.00452 | ± | 0.00461 | 0.12713 | ± | 0.03092 | 0.00342 | ± | 0.00237 | 0.00952 | ± | 0.00257 | 0.00084 | ± | 0.00039 | 0.08086 | ± | 0.02240 |
|  | Poly I:C-5 | 0.00551 | ± | 0.00176 | 0.14141 | ± | 0.01127 | 0.00635 | ± | 0.00160 | 0.00993 | ± | 0.00198 | 0.00116 | ± | 0.00026 | 0.09527 | ± | 0.01007 |
|  | Poly I:C-10 | 0.02206 | ± | 0.00937 | 0.17441 | ± | 0.04456 | 0.01533 | ± | 0.00655 | 0.01423 | ± | 0.00221 | 0.00129 | ± | 0.00024 | 0.14498 | ± | 0.03310 |
|  | Poly I:C-20 | 0.01946 | ± | 0.01343 | 0.15866 | ± | 0.05603 | 0.01441 | ± | 0.00970 | 0.01120 | ± | 0.00306 | 0.00126 | ± | 0.00047 | 0.13546 | ± | 0.06022 |
|  | RE | 0.13851 | ± | 0.09439 | 0.35018 | ± | 0.07166 | 0.05169 | ± | 0.03529 | 0.02929 | ± | 0.00915 | 0.00154 | ± | 0.00036 | 0.34764 | ± | 0.08462 |
