# Supplementary material for: Modeling for influenza vaccines and adjuvants profile for safety prediction system using gene expression profiling and statistical tools
Source: PLoS One. 2018 Feb 6;13(2):e0191896. doi: 10.1371/journal.pone.0191896 (PMC5800680; doi:10.1371/journal.pone.0191896)
Supplement: S9 Table — Data are presented as the mean ± S.D. (DOCX) [file pone.0191896.s010.docx]

**S9 Table**

The marker genes expression profiles in Poly I:C group

Data are presented as the mean ± S.D.

| Route | Vaccine and adjuvant | Marker genes | | | | | | | | | | | | | | | | | |
| --- | --- | --- | --- | --- | --- | --- | --- | --- | --- | --- | --- | --- | --- | --- | --- | --- | --- | --- | --- |
|  |  | *Lgals3bp* | | | *Zbp1* | | | *Mx2* | | | *Ifi47* | | | *Tapbp* | | | *Ifrd1* | | |
| ip | SA | 0.06451 | ± | 0.00458 | 0.00928 | ± | 0.00192 | 0.00374 | ± | 0.00012 | 0.04164 | ± | 0.00218 | 0.06328 | ± | 0.00358 | 0.13112 | ± | 0.01660 |
|  | HAv | 0.06480 | ± | 0.01063 | 0.00828 | ± | 0.00187 | 0.00464 | ± | 0.00079 | 0.03950 | ± | 0.00460 | 0.07301 | ± | 0.01715 | 0.15502 | ± | 0.01062 |
|  | Poly I:C-1 | 0.14808 | ± | 0.04749 | 0.03530 | ± | 0.02228 | 0.01748 | ± | 0.01145 | 0.06255 | ± | 0.02526 | 0.08208 | ± | 0.01399 | 0.13636 | ± | 0.02720 |
|  | Poly I:C-5 | 0.23765 | ± | 0.02362 | 0.09364 | ± | 0.01583 | 0.04104 | ± | 0.00684 | 0.12358 | ± | 0.01313 | 0.09247 | ± | 0.01233 | 0.16249 | ± | 0.01343 |
|  | Poly I:C-10 | 0.26305 | ± | 0.03794 | 0.09412 | ± | 0.01410 | 0.03998 | ± | 0.00382 | 0.11775 | ± | 0.00762 | 0.09467 | ± | 0.00603 | 0.14468 | ± | 0.00734 |
|  | Poly I:C-20 | 0.25564 | ± | 0.06709 | 0.12523 | ± | 0.05578 | 0.04195 | ± | 0.01353 | 0.13557 | ± | 0.04970 | 0.10624 | ± | 0.02933 | 0.14354 | ± | 0.00904 |
|  | RE | 0.53783 | ± | 0.03266 | 0.32592 | ± | 0.02677 | 0.11376 | ± | 0.00953 | 0.31020 | ± | 0.01208 | 0.15673 | ± | 0.00649 | 0.19966 | ± | 0.02339 |
|  |  |  |  |  |  |  |  |  |  |  |  |  |  |  |  |  |  |  |  |
| im | SA | 0.06571 | ± | 0.00369 | 0.01139 | ± | 0.00151 | 0.00460 | ± | 0.00091 | 0.03940 | ± | 0.00234 | 0.07119 | ± | 0.01075 | 0.12422 | ± | 0.00582 |
|  | HAv | 0.05732 | ± | 0.00716 | 0.00786 | ± | 0.00204 | 0.00351 | ± | 0.00096 | 0.03527 | ± | 0.00692 | 0.06199 | ± | 0.00626 | 0.13227 | ± | 0.00711 |
|  | Poly I:C-1 | 0.14029 | ± | 0.01894 | 0.03501 | ± | 0.00610 | 0.01313 | ± | 0.00338 | 0.06365 | ± | 0.00908 | 0.08381 | ± | 0.01252 | 0.13384 | ± | 0.01826 |
|  | Poly I:C-5 | 0.15148 | ± | 0.01917 | 0.03771 | ± | 0.00487 | 0.01756 | ± | 0.00272 | 0.06591 | ± | 0.00876 | 0.08068 | ± | 0.00843 | 0.13741 | ± | 0.01152 |
|  | Poly I:C-10 | 0.18094 | ± | 0.02619 | 0.05637 | ± | 0.01090 | 0.02363 | ± | 0.00171 | 0.08029 | ± | 0.00678 | 0.07425 | ± | 0.01045 | 0.12818 | ± | 0.01282 |
|  | Poly I:C-20 | 0.24338 | ± | 0.03101 | 0.08666 | ± | 0.00980 | 0.03500 | ± | 0.00498 | 0.10705 | ± | 0.00920 | 0.09254 | ± | 0.00748 | 0.13778 | ± | 0.01560 |
|  | RE | 0.51156 | ± | 0.11043 | 0.26829 | ± | 0.03938 | 0.08788 | ± | 0.01383 | 0.24235 | ± | 0.02913 | 0.15841 | ± | 0.04033 | 0.18896 | ± | 0.01993 |
|  |  |  |  |  |  |  |  |  |  |  |  |  |  |  |  |  |  |  |  |
| in | SA | 0.07434 | ± | 0.00696 | 0.01360 | ± | 0.00053 | 0.00501 | ± | 0.00129 | 0.04490 | ± | 0.00556 | 0.06411 | ± | 0.00956 | 0.12655 | ± | 0.00675 |
|  | HAv | 0.06756 | ± | 0.00606 | 0.01032 | ± | 0.00134 | 0.00411 | ± | 0.00043 | 0.03947 | ± | 0.00522 | 0.06367 | ± | 0.00972 | 0.13708 | ± | 0.00480 |
|  | Poly I:C-1 | 0.15739 | ± | 0.06502 | 0.04563 | ± | 0.03133 | 0.01552 | ± | 0.01086 | 0.06793 | ± | 0.02721 | 0.09086 | ± | 0.01848 | 0.13314 | ± | 0.01988 |
|  | Poly I:C-5 | 0.19336 | ± | 0.03143 | 0.05815 | ± | 0.01211 | 0.01715 | ± | 0.00253 | 0.07850 | ± | 0.00633 | 0.11358 | ± | 0.02786 | 0.14241 | ± | 0.01380 |
|  | Poly I:C-10 | 0.32166 | ± | 0.08105 | 0.13565 | ± | 0.04266 | 0.04139 | ± | 0.01579 | 0.11402 | ± | 0.02083 | 0.14839 | ± | 0.02876 | 0.16237 | ± | 0.02028 |
|  | Poly I:C-20 | 0.24757 | ± | 0.13290 | 0.10525 | ± | 0.06634 | 0.03256 | ± | 0.02024 | 0.09402 | ± | 0.03731 | 0.13907 | ± | 0.04893 | 0.14320 | ± | 0.02357 |
|  | RE | 0.55993 | ± | 0.07799 | 0.42167 | ± | 0.09062 | 0.18714 | ± | 0.06496 | 0.37326 | ± | 0.11460 | 0.27967 | ± | 0.05346 | 0.23148 | ± | 0.06208 |
