# Supplementary material for: Modeling for influenza vaccines and adjuvants profile for safety prediction system using gene expression profiling and statistical tools
Source: PLoS One. 2018 Feb 6;13(2):e0191896. doi: 10.1371/journal.pone.0191896 (PMC5800680; doi:10.1371/journal.pone.0191896)
Supplement: S10 Table — (DOCX) [file pone.0191896.s011.docx]

**S10 Table**

Ordinal logistic regression analysis for the average of the predicted outcomes in *i.p.* and *i.m.* or *i.p.* and nasal administered group.

|  |  |  | Analysis of Maximum Likelihood Estimation | | | | | | | | | | | | | | |
| --- | --- | --- | --- | --- | --- | --- | --- | --- | --- | --- | --- | --- | --- | --- | --- | --- | --- |
| Parameter | Whole-Model Test: Logit *r*^2^ |  | *β_0_* (REv) | | |  | *β_0_* (Poly I:C) | | |  | *β_1_* (ip) | | |  | *β_2_* (im or nasal) | | |
|  |  |  | Estimate | S.E. | *p* Value |  | Estimate | S.E. | *p* Value |  | Estimate | S.E. | *p* Value |  | Estimate | S.E. | *p* Value |
|  |  |  |  |  |  |  |  |  |  |  |  |  |  |  |  |  |  |
| ip *vs* im | 1 |  | -276.116 | 159586.710 | 0.99860 |  | -173.156 | 0.000 | 0.00010 |  | 277.236 | 0.000 | 0.00010 |  | 94.4762244 | 0 | 0.0001 |
| ip *vs* nasal | 1 |  | -248.269 | 122890.460 | 0.99840 |  | -161.941 | 0.000 | 0.00010 |  | 238.037 | 0.000 | 0.00010 |  | 37.4428297 | 0 | 0.0001 |
|  |  |  |  |  |  |  |  |  |  |  |  |  |  |  |  |  |  |
